# Citicoline Oral Solution Induces Functional Enhancement and Synaptic Plasticity in Patients with Open-Angle Glaucoma

**DOI:** 10.3390/jcm15010223

**Published:** 2025-12-27

**Authors:** Vincenzo Parisi, Lucia Ziccardi, Lucia Tanga, Lucilla Barbano, Emanuele Tinelli, Gianluca Coppola, Antonio Di Renzo, Manuele Michelessi, Gloria Roberti, Carmela Carnevale, Sara Giammaria, Carmen Dell’Aquila, Mattia D’Andrea, Gianluca Manni, Francesco Oddone

**Affiliations:** 1 IRCCS—Fondazione Bietti, Via Livenza 3, 00198 Rome, Italy; vincenzo.parisi@fondazionebietti.it (V.P.); lucia.tanga@fondazionebietti.it (L.T.); lucilla.barbano@fondazionebietti.it (L.B.); antonio.direnzo@fondazionebietti.it (A.D.R.); manuele.michelessi@fondazionebietti.it (M.M.); gloria.roberti@fondazionebietti.it (G.R.); carmela.carnevale@fondazionebietti.it (C.C.); sara.giammaria@fondazionebietti.it (S.G.); carmen.dellaquila@fondazionebietti.it (C.D.); francesco.oddone@fondazionebietti.it (F.O.); 2 Departmental Faculty of Medicine, UniCamillus-Saint Camillus International University of Health Sciences, 00131 Rome, Italy; 3 Department of Medicine and Health Sciences “V. Tiberio”, University of Molise, Via F. de Sanctis 1, 86100 Campobasso, Italy; 4 Department of Medical and Surgical Sciences, Magna Græcia University, 88100 Catanzaro, Italy; emanuele.tinelli@unicz.it; 5 Department of Medico-Surgical Sciences and Biotechnologies, Sapienza University of Rome Polo Pontino ICOT, 04100 Latina, Italy; gianluca.coppola@uniroma1.it; 6 Department of Sense Organs, Faculty of Medicine and Dentistry, Sapienza University of Rome, Viale del Policlinico 155, 00161 Rome, Italy; mattia.dandrea@uniroma1.it (M.D.); gianlucamanni53@gmail.com (G.M.)

**Keywords:** glaucoma, PERG, VEP, Citicoline, synaptic plasticity

## Abstract

**Objectives:** To evaluate the changes in retinal function and neural conduction along the visual pathways after 12 months of treatment with Citicoline oral solution in patients with open-angle glaucoma (OAG). **Methods:** In this randomized, prospective, double-blind study, 29 OAG patients were enrolled. Fifteen patients (Citicoline Group, 15 eyes) received Citicoline oral solution (Neurotidine^®^, 500 mg/day), and 14 patients (Placebo Group, 14 eyes) received placebo for 12 months. Visual field (VF), pattern electroretinogram (PERG), visual evoked potentials (VEP), and Retinocortical Time (RCT) were assessed at baseline and after 6 and 12 months. Brain Diffusion Tensor Imaging (DTI)-Magnetic Resonance Imaging (MRI) was performed at baseline and at 12 months. **Results:** PERG, VEP, and RCT baseline values were comparable between groups (*p* > 0.01) at baseline. After 12 months of Citicoline treatment, significant (*p* < 0.01) increases in PERG P50–N95 and VEP N75-P100 amplitudes, and significant shortening of PERG P50, VEP P100 implicit times and RCT were observed. VEP implicit times shortening significantly correlated with the changes in VF Mean Deviation, and RCT shortening was associated with changes in DTI-MRI metrics in the lateral geniculate nucleus and on optic radiations, without reaching the level of significance. No significant changes were found in the Placebo Group. **Conclusions:** In OAG, Citicoline oral solution enhances retinal function likely through neuromodulation processes and changes post-retinal visual pathway connectivity. This could explain the improvement of visual field defects.

## 1. Introduction

Open-angle glaucoma (OAG) is a chronic, progressive neurodegenerative disease that leads to the progressive loss of retinal ganglion cells (RGCs) and to structural alterations of the optic nerve head [1]. Its most common variant, primary open-angle glaucoma, is primarily associated with increased intraocular pressure (IOP), which remains the main target of therapy through IOP-lowering medications, laser, or surgery. Nonetheless, in over a third of patients, visual function continues to deteriorate despite well-controlled IOP, suggesting that mechanisms independent from IOP may also play a significant role in disease progression [2].

An initial mechanical or vascular insult compromises blood supply to the optic nerve head and disrupts anterograde and retrograde axonal transport of essential metabolites and neurotrophic factors, leading to RGC apoptosis [3]. A secondary insult creeps in: the glutamate-mediated excitotoxicity. In this phase, excessive glutamate release from apoptotic neurons overstimulates NMDA receptors, leading to abnormal Ca++ influx into surrounding neurons, initiating a biochemical cascade culminating in further apoptosis [4,5]. Additionally, the overactivation of phospholipase A2 contributes to membrane degradation through the breakdown of phosphatidylcholine, further exacerbating cell death [6].

Electrophysiological methods (simultaneous recordings of Pattern Electroretinogram, PERG, and Visual Evoked Potentials, VEP) allow direct functional evaluation of RGCs, of the neural conduction along the small and large axons along the visual pathways and to obtain an electrophysiological index [Retinocortical Time (RCT)] of the post-retinal neural conduction [7].

In OAG patients, abnormal PERG and VEP responses and delayed RCT were detected, leading to the hypothesis that visual field deficits in glaucoma result from dual mechanisms: one at the level of the RGCs and the other one involving the post-retinal optic pathways. The latter has been ascribed to abnormal synaptic circuitry driven by diminished neural input from RGCs to the lateral geniculate nucleus (LGN), ultimately affecting cortical activity of the occipital lobe, with consequent visual field deficits [7]. In neuroimaging studies, which have documented structural changes in several key components of the visual system. In glaucoma eyes, neuronal loss has been observed at the level of the LGN [8], optic tract [9], and visual cortex [10,11,12,13,14]. Moreover, these alterations extend beyond anatomical damage. Indeed, functional Magnetic Resonance Imaging (MRI) has shown disrupted connectivity within not only visual but also cognitive networks, including those responsible for attention and executive function [14,15]. These neuro-radiological findings reinforce the notion that glaucoma, though classically seen as an ocular disease, is characterized also by widespread neurodegenerative processes affecting the brain.

Considering all this and observing that, in several OAG patients, IOP lowering is not sufficient to halt the progression of visual impairment [2], new active strategies, such as the enhancement of RGC function (neuroenhancement) and neuroprotection [16], have been suggested to slow down the progression rate of glaucomatous damage.

Several putative neuroprotective agents have been tested in these years. These include Memantine, Brimonidine, Coenzime Q10, recombinant human nerve growth factor, Nicotinamide, and Citicoline. Despite preclinical study results, many of these approaches fail to reach clinical proof of evidence [17].

In this context, the neuroprotective and neuromodulatory role of Citicoline in glaucoma emerged, thanks to the multifactorial mechanism of action previously described (i.e., preservation of cardiolipin and sphingomyelin, restoration of phosphatidylcholine, stimulation of glutathione synthesis, lowering of glutamate concentration, rescuing mitochondrial function, and a dopaminergic-like action [18], stimulating action on proteasome activity [19]). Since 1998, several studies have demonstrated that Citicoline enhances RGC function [20], improves the neural conduction along the visual pathways [20,21], and reduces the retinal nerve fiber layer (RNFL) loss [20] in glaucoma patients. Ultimately, Citicoline oral solution and eye drops showed to slow down visual field deterioration and RNFL thinning [20] and to improve the quality of vision [22] in OAG.

To date, no previous studies have yet investigated whether Citicoline could induce improvement on the post-retinal neural conduction by electrophysiological tests and whether these functional ameliorations can be accompanied by measurable structural and functional changes at the level of LGN, optic radiations or the visual cortex [as evaluated by Brain Diffusion Tensor Imaging (DTI)-MRI].

The aim of this trial was twofold. The primary objective was to evaluate whether, in OAG eyes, Citicoline oral solution, a formulation with high bioavailability, administered for 12 months, could improve RGC function and the post-retinal neural conduction along both large and small axons forming the visual pathways (as measured by the changes in PERG and in VEP responses) and whether these functional changes should be related between them and to the visual field variations. The secondary objective was to assess whether Citicoline oral solution could improve the above-mentioned abnormal synaptic circuitry involving both the RGC axons and the LGN, with consequent functional changes (as measured by RCT changes) and/or structural modifications in the post-retinal visual pathways (LGN, optic radiations, visual cortex), as evaluated by DTI-MRI.

Through this multimodal approach, the study seeks to clarify the interactions between retinal and post-retinal components of the visual system in glaucoma and to define the extent to which Citicoline could influence both.

## 2. Materials and Methods

### 2.1. Participants

A total of thirty-two patients affected by OAG were selected from a cohort of 230 individuals, based on specific inclusion criteria.

- Inclusion criteria were as follows: visual field deficit, assessed using the Humphrey Field Analyzer (HFA) 24-2 program, with a Mean Deviation (MD) ranging from −6 to −25 dB. Test reliability was ensured by including only examinations with fixation losses, false positives, and false negatives each below 20% [23]; best-corrected visual acuity (BCVA) between 0.0 and 0.1 logMAR;
- Characteristic signs of glaucomatous optic nerve damage as documented by color stereo-photographs and corresponding visual field defects. Optic disk photos and visual fields were independently reviewed by two expert glaucoma specialists (FO and LT). In case of disagreement, a third glaucoma specialist (MM) adjudicated, and the final classification was assigned by majority vote; Refractive error between −3.00 and +3.00 spherical equivalent;
- Absence of any history or documented evidence of diseases affecting the cornea, lens, macula, or retina as well as absence of diabetes, optic neuritis, or any neurological conditions involving the visual pathways; Pupil diameter greater than 3 mm without the use of mydriatics;
- Central corneal thickness within 500–600 µm, as measured using ultrasonic pachymetry (AL 2000 Bio & Pachymeter, Tomey Corporation, Nagoya, Japan); beta-blocker monotherapy (with IOP < 18 mmHg) maintained consistently for at least eight months prior to baseline evaluation—and throughout the study—since PERG responses can be affected by pharmacological IOP reduction [24,25,26,27].

Based on the above-mentioned inclusion criteria, 32 patients were selected, 16 patients randomized for each group (see below). We considered 32 OAG eyes from 32 patients; when both eyes met inclusion criteria, only one eye per patient was included in the study. The selected eye was the one exhibiting greater RGC dysfunction, as indicated by lower PERG P50–N95 amplitude values (see Section 2.3).

### 2.2. Study Design

This was a randomized, prospective, double-blind study. The protocol adhered to the Declaration of Helsinki and was approved on 16 November 2021 by the Local Ethics Committee (Comitato Etico Centrale IRCCS Lazio, protocol number 1628/21/FB). All patients were informed on the study outcomes and gave written informed consent prior to participation.

At baseline, the 32 selected patients were randomly assigned to two age-similar groups of 16 individuals each: the Citicoline Group (mean age 59.8 ± 4.32 years, mean IOP 13.567 ± 2.019 mmHg) and the Placebo Group (mean age 60.2 ± 5.12 years, mean IOP 12.988 ± 2.207 mmHg). Randomization, performed using an electronic system (https://www.randomizer.org/, last access on 6 June 2024), was stratified according to age, sex, IOP, HFA MD, and VEP P100 implicit time. Allocation remained masked to all investigators until the completion of the follow-up period.

From baseline to month 12, both groups continued treatment with topical beta-blockers. In addition, the Citicoline Group received 10 mL/day of Neurotidine^®^ (oral solution containing citicoline free acid 500 mg/10 mL, water, fructose, sodium citrate, sodium hydroxide, potassium sorbate, and riboflavin). The Placebo Group received daily the vehicle solution without Citicoline.

Adherence to treatment was assessed via questionnaires distributed at each visit asking patients to self-grade their adherence as good or poor if they missed, respectively, less or more than 30% of doses. Poor adherence was considered a criterion for excluding the patients from the study.

### 2.3. Electrophysiological (PERG and VEP) Assessment

PERG and VEP recordings were performed simultaneously at baseline, 6 months, and 12 months in both Citicoline and Placebo Groups using the Retimax Advanced Plus system (CSO, Florence, Italy), in accordance with previously described methodologies [7].

Visual stimulation was monocular, with the fellow eye occluded. Stimuli consisted of high-contrast (80%) checkerboard patterns presented on a monitor with a mean luminance of 110 cd/m^2^, reversing at 2 Hz. At a viewing distance of 114 cm, check sizes subtended either 60 min or 15 min of arc, as recommended by ISCEV standards [28] to preferentially stimulate large and small axons, respectively. The monitor subtended a total visual angle of 23°, and a central fixation target (approximately 0.5°) was provided. All patients confirmed that the fixation target was clearly visible.

PERG signals were recorded via Ag/AgCl skin electrodes placed on the lower eyelid. A bipolar configuration was used, with the active electrode on the stimulated eye and the reference electrode on the patched eye. The P50 and N95 components were identified, and the P50 implicit time (PERG IT) and peak-to-peak P50–N95 amplitude (PERG A) were measured.

For VEP responses, the N75 and P100 components were analyzed, the P100 implicit time (VEP IT) and peak-to-peak N75-P00 amplitude (VEP A) were measured. The Retinocortical Time (RCT) was calculated as the difference between VEP P100 and PERG P50 implicit times [7].

Each recording session included a minimum of two and a maximum of six repeated acquisitions to verify waveform reproducibility. Based on previous findings [7], intra-individual variability was estimated as ±2 ms for VEP P100 implicit time and ±0.18 µV for PERG amplitude. Two recordings were considered reproducible if the difference fell within these ranges. When necessary, additional recordings were performed, never exceeding six per session. In addition, the two reproducible recordings provided the data for the test–retest intra- and inter-individual variability of the entire cohort of enrolled patients.

For statistical analysis, the averaged values of PERG and VEP parameters from reproducible traces were considered.

Signal-to-noise ratio (SNR) for PERG and VEP responses was assessed using previously established methods [7]. Only recordings with SNR values greater than 2 were included in the analysis.

### 2.4. MRI Protocol

#### 2.4.1. DTI Acquisition

DTI was performed using a single-shot echo-planar imaging sequence.

Acquisition parameters included a repetition time (TR = 12,700 ms) echo time (TE: 109.1 ms; voxel 3 × 3 × 3 mm^3^, no gap), matrix size 96 × 96. Each dataset included 30 diffusion directions with a b-value of 1000 s/mm^2^ and one b = 0 volume in both Anterior–Posterior (AP) and Posterior–Anterior (PA) phase-encoding directions. Additionally, T1-weighted 3D anatomical images (TR = 13.3 ms; TE = 4.2 ms; voxel 1 × 1 × 1 mm^3^ no gap; matrix 256 × 256) and T2-weighted Turbo Spin Echo sequence were acquired (TR = 3956 ms; TE = 92.5 ms; voxel 5 × 0.8 × 1.3 mm^3^; gap = 1 mm; matrix 320 × 192).

#### 2.4.2. DTI Preprocessing and TBSS Analysis

All DTI datasets were visually inspected by a trained neuroradiologist (E.T.) to identify motion artifacts, signal dropout, or cardiac pulsation effects. Preprocessing was carried out using the FMRIB Software Library (FSL, version 6.0.7; https://fsl.fmrib.ox.ac.uk/fsl; last access on 26 June 2025) [29,30,31]. The topup tool was applied to correct for susceptibility-induced distortions using paired b = 0 volumes acquired in AP and PA and Posterior directions [32]. Brain extraction was performed using the brain extraction tool algorithm [33,34], and motion and eddy-current correction was achieved with the eddy tool.

The DTIFIT (Diffusion tensor imaging fit) toolbox was then used to compute fractional anisotropy (FA), mean diffusivity (MDi), axial diffusivity (AD), and radial diffusivity (RD) maps from the corrected diffusion volumes, associated b-values, vectors, and brain masks.

Regions of interest (ROIs) corresponding to the geniculate nuclei [35] and optic radiations were defined using FSLeyes, aligned to the MNI152 T1 template, and registered to individual diffusion space using tools from the FDT suite (version 1.17.0.dev19) [36,37]. Participants’ DTI metrics for each ROI were extracted for statistical evaluation and descriptive statistics were stored (mean, std and 95% confidence interval).

Voxel-wise statistical analysis of FA was performed using tract-based spatial statistics (TBSS) [38]. FA images were nonlinearly registered to a 1 mm isotropic FMRIB58_FA template using FNIRT (version v1.2). A mean FA image was computed, skeletonized (FA threshold > 0.2), and individual FA maps were projected onto the common skeleton. The same procedure was used for MDi, AD, and RD analyses. Group comparisons were conducted using FSL’s randomize tool (version 2.9) employing non-parametric permutation testing for both cross-sectional and longitudinal comparisons.

### 2.5. Statistical Analysis

The primary endpoint was the change in VEP P100 implicit time in the Citicoline Group relative to the Placebo Group. Sample size estimation was based on previous data [39], which reported a baseline VEP P100 latency of 128.5 ± 7.45 ms and a post-treatment latency of 120.4 ± 7.41 ms after Citicoline eye drops. Assuming an alpha error of 5% and a power of 80%, nine subjects per group were required. Considering a dropout rate of 30%, the final target was adjusted to 12 participants per group.

Descriptive statistics showed mean values, standard deviations, 95% confidence intervals, and effects sizes of Cohen’s d dimension for each parameter.

Differences in PERG and VEP responses between groups were analyzed using one-way ANOVA and Dunnett’s method to correct for multiple comparisons in longitudinal studies, while Tukey’ method for cross-sectional studies was also used. A *p*-value less than 0.05 was considered statistically significant.

To improve normality, electrophysiological data were logarithmically transformed before analysis.

Pearson’s correlation coefficients were calculated to evaluate associations between changes in PERG/VEP, visual field MD, and MRI metrics and a *p*-value less than 0.01 was considered statistically significant.

SPSS (version 25), MedCalc V.13.0.4.0 (MedCalc, Mariakerke, Belgium) and R (V.4.3.1) were used for statistical analysis.

## 3. Results

One patient from the Citicoline Group and two from the Placebo Group were excluded due to poor compliance, resulting in 15 and 14 patients, respectively, completing the full 12-month protocol design.

The IOP did not show statistically significant (*p* > 0.01) changes throughout the entire follow-up of the study in both groups.

Both Citicoline oral solution and Placebo were well tolerated with no reported adverse events throughout the study.

### 3.1. PERG Data

As shown in Table 1, at baseline, there were no significant differences (*p* > 0.01) in 60′ and 15′ PERG IT and PERG A values in Citicoline and Placebo Groups.

**Figure 1:**
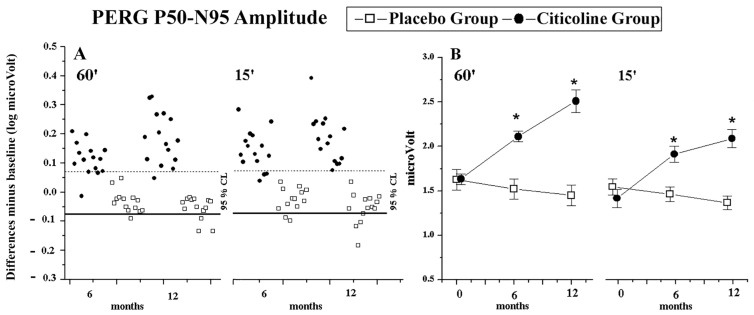
PERG P50–N95 Amplitude individual changes (**A**) and graphical representation of mean values (**B**) observed in OAG patients treated with beta-blocker monotherapy plus Citicoline oral solution (Citicoline Group, 15 eyes) and in OAG patients treated with beta-blocker monotherapy plus Placebo (Placebo Group, 14 eyes). The percentage of unmodified eyes (within the 95% confidence test–retest limit, CL), eyes with improvement (values over the 95% confidence test–retest limit—dashed line), and eyes with worsening (values under the 95% confidence test–retest limit for amplitudes—solid line) is reported in Table S1. The statistical changes for PERG amplitude values are reported in Table 1. Vertical lines: one error standard. * Indicates ANOVA versus baseline, *p* < 0.01. 60′ and 15′ refer to visual stimuli in which each check subtended 60 min and 15 min of the visual arc, respectively.

Considering the individual differences observed at 6 and 12 months of follow-up with respect to baseline, the majority of patients of the Citicoline Group (range from 73.33% to 92.86%) showed an improvement of the values of PERG ITs and PERG As, whereas the majority of patients belonging to the Placebo Groups (range from 78.57% to 92.86%) presented unchanged values of PERG ITs and PERG As (see Table S1 and Figure 1A).

When considering the mean changes detected at 6 and 12 months minus baseline, significant differences (*p* < 0.01) in PERG IT and PERG A values between Citicoline and Placebo Groups were observed (see Table S2).

On average, as reported in Table 1 and in Figure 1B, in Citicoline Group a significant (*p* < 0.01) shortening of 60′ and 15′ PERG IT values and a significant (*p* < 0.01) increase in 60′ and 15′ PERG A values were detected at 6 and 12 months with respect to baseline values. In the Placebo Group, significant (*p* < 0.01) further delay of 60′ PERG ITs at 12 months with respect to baseline was found. During the follow-up (6 and 12 months), no other significant differences were observed.

### 3.2. VEP Data

At baseline, no significant differences (*p* > 0.01) in 60′ and 15′ VEP IT and VEP A values were observed in Citicoline and Placebo Groups.

**Figure 2:**
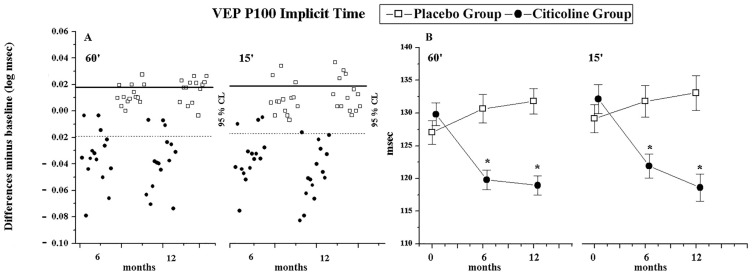
VEP P00 Implicit Time individual changes (**A**) and graphical representation of mean values (**B**) observed in OAG patients treated with beta-blocker monotherapy plus Citicoline oral solution (Citicoline Group, 15 eyes) and in OAG patients treated with beta-blocker monotherapy plus Placebo (Placebo Group, 14 eyes). The percentage of unmodified eyes (within the 95% confidence test–retest limit, CL), eyes with improvement (values under the 95% confidence test–retest limit—dashed line), and eyes with worsening (values over the 95% confidence test–retest limit—solid line) is reported on Table S1. The statistical changes for VEP P100 Implicit Time values are reported in Table 2. Vertical lines: one error standard. * Indicates ANOVA versus baseline, *p* < 0.01. 60′ and 15′ refer to visual stimuli in which each check subtended 60 min and 15 min of the visual arc, respectively.

When the individual differences (6 and 12 months of follow-up with respect to baseline) were considered, the majority of patients belonging to the Citicoline Group (range from 53.33% to 93.33%) showed an improvement of VEP parameters; by contrast, the majority of patients belonging to the Placebo Group (range from 78.57% to 92.86%) showed substantial unchanged values of VEP parameters (see Table S1 and Figure 2A).

Table S2 reports the mean values of individual differences detected at 6 and 12 months minus baseline and the significant differences in VEP IT and VEP A. Significant (*p* < 0.01) differences between Citicoline and Placebo Groups were found.

As presented in Table 2 in the Citicoline Group, significant (*p* < 0.01) shortening of 60′ and 15′ VEP ITs (see Figure 2B) and significant (*p* < 0.01) increase in 60′ and 15′ VEP As were detected at 6 and 12 months with respect to baseline values. In the Placebo Group, during the follow-up (6 and 12 months), no significant (*p* > 0.01) differences in VEP ITs and with respect to baseline values were found.

### 3.3. Retinocortical Time Data

**Figure 3:**
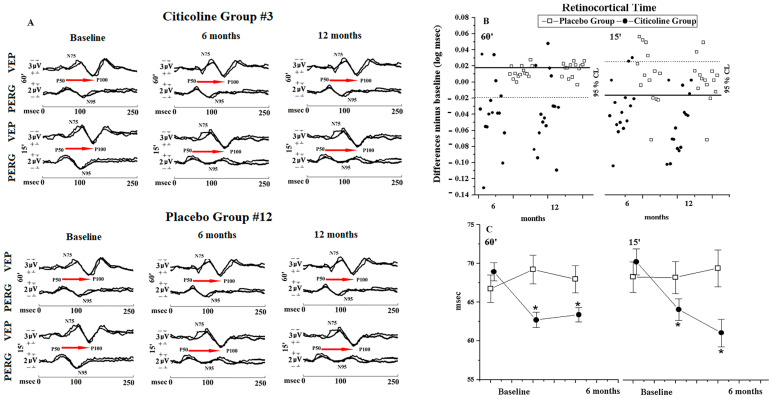
(**A**) Examples of simultaneous VEP and PERG recordings performed in one OAG eye treated with beta-blockers plus Citicoline oral solution (Citicoline Group #3) and in one OAG eye treated with beta-blocker monotherapy plus Placebo (Placebo Group #12) at baseline (baseline), and after 6 and 12 months of treatment. The red arrow (→) indicates the Retinocortical Time (RCT, difference between VEP P100 and PERG P50 Implicit Times). In comparison with the baseline condition, after treatment with Citicoline oral solution, increase in PERG and VEP amplitudes and shortening of VEP and PERG implicit times and of RCT were detected. An increase in VEP amplitudes, together with changes in the visual field defects, can also be observed. After treatment with Placebo, PERG and VEP Implicit Times and Amplitudes and RCT were similar to baseline ones. (**B**) Plot of individual changes and (**C**) graphical representation of mean values observed in OAG patients treated with beta-blocker monotherapy plus Citicoline oral solution (Citicoline Group, 15 eyes) and in OAG patients treated with beta-blocker monotherapy plus Placebo (Placebo Group, 14 eyes). The percentage of unmodified eyes (within the 95% confidence test–retest limit, CL), eyes with improvement (values under the 95% confidence test–retest limit—solid line), and eyes with worsening (values over the 95% confidence test–retest limit for amplitudes—dashed line) is reported on Table S1. The statistical changes for RCT values are reported in Table 3. Vertical lines: one error standard. *: ANOVA versus baseline, *p* < 0.01. 60′ and 15′ refer to visual stimuli in which each check subtended 60 min and 15 min of the visual arc, respectively.

Figure 3A shows examples of simultaneous PERG and VEP recordings and relative RCT performed in one OAG patient treated with beta-blocker monotherapy and additional treatment with Citicoline oral solution for 12 months (Citicoline Group #3) and in one OAG patient treated with beta-blocker monotherapy and additional treatment with Placebo for 12 months (Placebo Group #12).

At baseline, Citicoline and Placebo Groups showed similar values (*p* > 0.01) of 60′ and 15′ RCT (see Table 3).

Considering the individual differences (6 and 12 months of follow-up with respect to baseline), most of the Citicoline Group patients (ranging from 73.33% to 80.00%) showed an improvement of RCT, whereas many Placebo Group patients (ranging from 42.86% to 71.43%) showed unmodified RCT values (see Table S1 and Figure 3B).

Table S2 presents the mean values of individual differences detected at 6 and 12 months minus baseline. During both 6- and 12-months follow-up, significant (*p* < 0.01) differences in RCT between Citicoline and Placebo Groups were found.

As shown in Table 3 and in Figure 3C, in the Citicoline Group a significant (*p* < 0.01) shortening of RCT values with respect to baseline was found at 6 and 12 months of treatment. In the Placebo Group, during the follow-up (6 and 12 months), no significant (*p* > 0.01) differences in RCT values with respect to baseline ones were observed.

### 3.4. Correlations Between PERG and VEP Data

**Figure 4:**
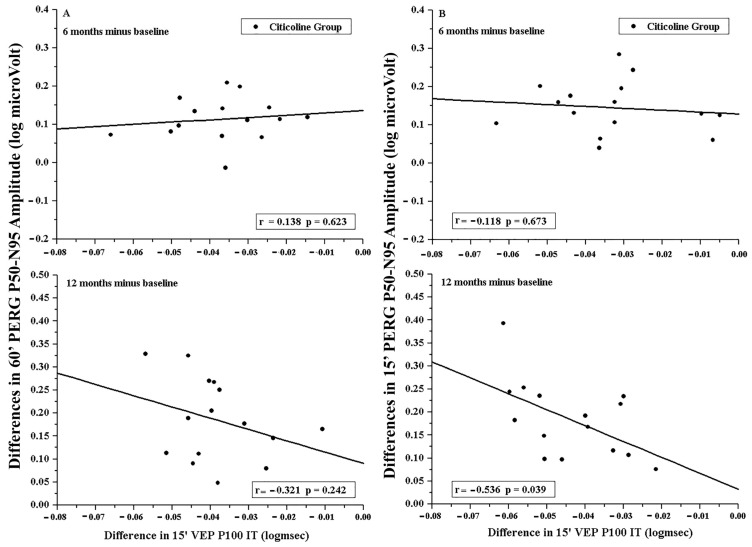
Individual differences (6 and 12 months minus baseline) in PERG P50–N95 amplitude observed in Citicoline Group plotted as a function of the corresponding differences in VEP P100 Implicit Time. 60′ (**A**) and 15′ (**B**) refer to visual stimuli in which each check subtended 60 min and 15 min of the visual arc, respectively.

As presented in Figure 4, in the Citicoline Group, no significant (*p* > 0.01) linear correlations between the individual differences (both 6 and 12 months minus baseline) of PERG As and VEP ITs were detected.

### 3.5. Visual Field Data and Correlation with PERG and VEP Data

At baseline, the HFA 24-2 MD values in the Citicoline Group (−14.805 ± 5.886 dB) were not significantly (*p* > 0.01) different with respect to those of the Placebo Group (−12.816 ± 3.815 dB).

In the Citicoline Group, based on the test–retest MD values (±0.48 dB), at 6-month follow-up, an MD variation greater than +0.48 dB was detected on 11/15 eyes (73.33%); a mean change of 1.641 ± 1.369 dB was found. At 12-month follow-up, an MD change greater than +0.48 dB was detected on 12/15 eyes (80.00%), with a mean change of 1.841 ± 1.658 dB. The MD mean changes observed in the Citicoline Group were significantly different (*p* < 0.01) (6 months: f = 18.94, *p* < 0.01; 12 months: f = 19.87, *p* < 0.01) than those found in the Placebo Group (6 months: −0.409 ± 1.148 dB; 12 months: −0.471 ± 1.042 dB).

At baseline, the HFA 24-2 Pattern Standard Deviation (PSD) values were not significantly (*p* > 0.01) different between Citicoline (10.116 ±3.313 dB) and Placebo Groups’ ones (10.594 ± 2.968 dB).

At 6 and 12 months of follow-up, considering the test–retest PSD values (±0.39 dB), in the Citicoline Group, PSD changes were observed on 10/15 (66.66%) and 12/15 (80.00%); mean changes of −1.014 ± 1.301 dB and −1.017 ± 1.266, respectively, were found. The PSD mean changes observed in the Citicoline Group were significantly different (6 months: f = 8.87, *p* < 0.01; 12 months: f = 8.82, *p* < 0.01) with respect to the Placebo Group ones (6 months: 0.481 ± 1.402 dB; 12 months: 0.493 ± 1.488 dB).

**Figure 5:**
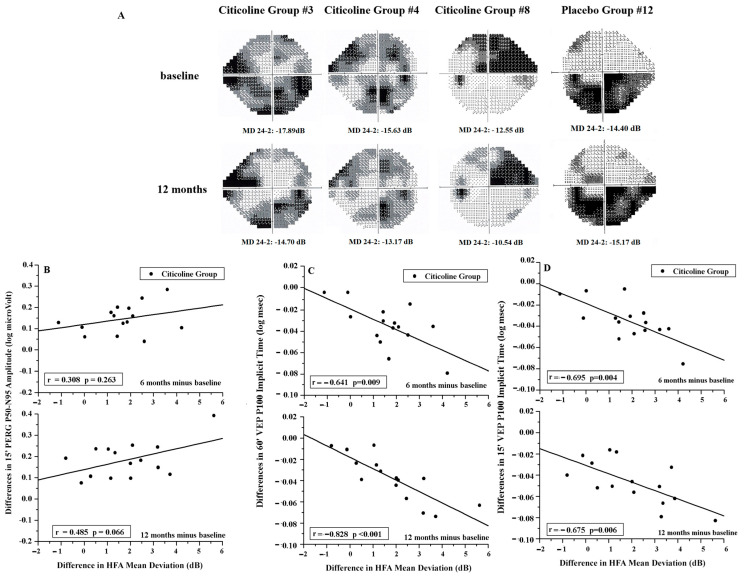
(**A**) Examples of Humphrey Field Analyzer 24-2 (HFA) at baseline and after 12 months of treatment with Citicoline oral solution (Citicoline Group #3, Citicoline Group #4, Citicoline Group #8) or Placebo (Placebo Group #12). It should be noted that in the 3 OAG patients of the Citicoline Group, at the end of treatment with Citicoline oral solution, reductions in Mean Deviation (MD) of 3.19, 2.46, and 2.01 dB were detected, respectively. In the patient of the Placebo Group, at the end of treatment with placebo, an increase in Mean Deviation of 0.17 dB was observed. (**B**) Individual differences (6 and 12 months minus baseline) in PERG P50–N95 amplitude observed in Citicoline Group plotted as a function of the corresponding differences in HFA Mean Deviation. (**C**,**D**) Individual differences (6 and 12 months minus baseline) in VEP Implicit Times observed in Citicoline Group plotted as a function of the corresponding differences in HFA Mean Deviation. 60′ and 15′ refer to visual stimuli in which each check subtended 60 min and 15 min of the visual arc, respectively.

As shown in Figure 5B–D, in the Citicoline Group, no correlations (*p* > 0.01) were found between the changes in PERG As and in MD at both 6 and 12 months of follow-up; on the contrary, the reduction in MD was significantly correlated (*p* < 0.01) with the shortening of VEP ITs at both 6 and 12 months of follow-up.

### 3.6. MRI Data and Correlation with PERG and VEP Data

As reported in Table 4, at baseline, Citicoline and Placebo Groups showed similar values of all DTI parameters (FA, MDi, AD, RD) on both LGN and optic radiations evaluation.

In the Citicoline Group, the observed mean values of DTI metrics were modified with respect to baseline and in particular an increase in FA and AD and a reduction in MDi and RD were found. Nevertheless, during the follow-up (12 months), no significant (*p* > 0.01) differences in all DTI metrics values with respect to baseline values were found. In the Placebo Group, no significant (*p* > 0.01) differences were observed at 12 months versus baseline for all DTI metrics.

In the Citicoline Group, the individual changes in DTI metrics (increase in FA and AD and a reduction in MDi and RD) detected in both left and right optic radiation and LGN were associated with the observed shortening of RCT, without reaching the level of significance (ranging from r = 0.391, *p* = 0.081 for AD optic radiations right vs. RCT to r = 0.551, *p* = 0.054 for RD LGN right vs. RCT).

**Figure 6:**
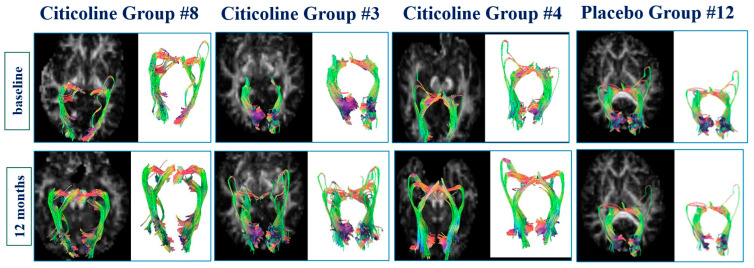
Example of diffusion tensor fiber tractography renderings of the optic radiations in three patients of Citicoline Group and in one patient of Placebo Group at baseline and after 12 months of treatment.

In Figure 6, a representative example of DTI is reported in three patients of the Citicoline Group and in one patient of the Placebo Group at baseline and after 12 months of treatment.

## 4. Discussion

The aim of the present study was to assess whether in OAG patients the treatment with Citicoline oral solution, administered for 12 months, could improve the function of RGCs (as measured by the changes in PERG responses) and the neural conduction along both large and small axons forming the visual pathways (as measured by the changes in VEP responses) with consequent improvement of visual field defects. In addition, our purpose was to evaluate whether Citicoline oral solution could change the microstructure at the level of synaptic circuitries of post-retinal visual pathways (as measured by RCT and DTI-MRI changes).

We disclaim also that patients treated with Citicoline oral solution did not report any adverse events throughout the study. No patient discontinued the study because of side effects linked to trial products (i.e., Citicoline and Placebo). According to previous evidence [22], this treatment can be considered safe and well tolerated.

### 4.1. Effect of Citicoline Oral Solution on RGC Function (PERG Evidence)

In the present study, we evaluated the RGC function by PERG recordings by using visual stimuli (60′ and 15′ of visual arc) different from those suggested by ISCEV standards [40]. In particular, we used 15′ of visual stimuli, since it is reported that this type of pattern stimulation has a sensitivity and a specificity of 100% in OAG patients to detect RGC dysfunction [41].

Furthermore, PERG has been shown to be an optimal tool for the early detection of RGC dysfunction in glaucoma, being able to identify it before manifesting glaucomatous visual field damage [41].

In our cohort of OAG patients, after treatment with Citicoline oral solution, significant shortening of PERG ITs and an increase in PERG As were found. This agrees with other previous studies with Citicoline [20]. In this case, Citicoline was administered in oral solution, since it is well known that this type of pharmacological administration has a bioavailability of 98% [42,43].

By contrast, in the Placebo Group, a significant increase in PERG 15′ IT was observed, suggesting further RGC dysfunction after 12 months of follow-up despite non-significant changes in IOP in this group. All this could suggest a progression of the RGC impairment and it is in contrast with previous evidence showing no significant PERG 15′ IT changes in OAG patients treated with beta-blockers+Placebo or exclusively with beta-blockers during periods of 180 and 360 days [44,45]. This discrepancy can be ascribed to the different studied population, since the HFA MD ranged between −2 and −6 dB in the reports with no significant changes in PERG 15′ IT [44,45], whereas in our study, HFA MD ranged between −6 and −25 dB.

The multifactorial mechanisms of action of Citicoline leading to the improvement of RGC function have been extensively reviewed by Faiq et al. [18] and Oddone et al. [20]. Briefly, Citicoline exerts its action through its role as an endogenous intermediate in phosphatidylcholine biosynthesis. It contributes to the maintenance of phospholipid membrane and redox homeostasis; moreover, it supports mitochondrial dynamics and the modulatory effect on the dopaminergic neurotransmission. As a precursor of phospholipidic membrane components, such as phosphatidylcholine and sphingomyelin, Citicoline also sustains neuronal membrane integrity. Enhancing sphingomyelin synthesis, and stabilizing RGC axonal membranes, limiting free fatty acid release, it mitigates redox imbalance and neuroinflammatory responses. Additionally, Citicoline promotes mitochondrial function.

About the direct effect of Citicoline on the RGCs, in two experimental models of cultured retina, it was observed that the RGC degeneration can be reduced by applying Citicoline [46,47]. In addition, Matteucci et al. [48] observed that in retinal cultures treated with glutamate or high glucose (a model of neurodegeneration), the administration of Citicoline induces a reduction in pro-apoptotic effects of the synaptic loss.

In summary, the observed effect of Citicoline oral solution improving the RGC function of OAG patients can be ascribed to above-mentioned different but concomitant factors [18,19,20].

We are aware that no changes in PERG P50–N95 amplitude in OAG patients under Citicoline treatment, administered in different modalities (intramuscularly, orally and topically), have been found in a previous metanalysis regarding the effect of Citicoline on glaucoma progression [49].

### 4.2. Effect of Citicoline Oral Solution on Neural Conduction Along the Visual Pathways (VEP Evidence)

In our study, we assessed the neural conduction along the visual pathways by pattern VEP recordings in response to checks subtaining 60′ and 15′ of visual arc, following the ISCEV standard [28]. We used both 60′ and 15′ of visual stimuli, and it is worth noting that when VEP is recorded in response to the 15′ pattern stimuli, a sensitivity and specificity of 100% and a significant correlation with visual field HFA MD have been found in OAG patients, suggesting a correlation between the visual pathways dysfunction and the psychophysical responses [41].

A significant shortening of VEP ITs and an increase in VEP after treatment with Citicoline oral solution was observed.

With respect to previous studies [20,39], in which it was observed that Citicoline improves the neural conduction along the small axons (in fact, VEP recorded exclusively in response to checks subtaining 15′ of visual arc assesses the neural conduction along the small axons [7]), it is worth noting that an element of novelty of the present study is represented by the evaluation of the neural conduction along the large axons forming the visual pathways (indeed, VEP is recorded in response to checks subtaining 60′ of visual arc) after treatment with Citicoline oral solution. It was interesting to observe an improvement (shortening in ITs and increase in As) of 60′ VEP responses leading to the conclusion that Citicoline oral solution may improve the neural conduction not only of the small axons (improvement of 15′ VEP) but also of the large axons (improvement of 60′ VEP).

When OAG patients were treated with Citicoline eye drops, based on the correlation between the increase in PERG responses and the shortening of VEP responses, it was suggested that the administration of Citicoline reaching the neural retinal elements induces a reduction in RGC dysfunction with consequent improvement of the neural conduction along the visual pathways [39].

This effect was not observed in our cohort of OAG patients: no significant correlation between the increase in PERG responses and the shortening of VEP responses was found. All this leads us to believe that with Citicoline oral solution, the improvement of the neural conduction along both small and large axons is independent from the reduced RGC dysfunction and therefore other factors (see below, point 4.3) must be considered.

### 4.3. Effect of Citicoline Oral Solution on Post-Retinal Visual Pathways (RCT and DTI Evidence)

In our cohort of OAG patients, the effects of Citicoline oral solution on neural conduction along large and small axons of the post-retinal visual pathways was estimated by evaluating the changes in RCT, which is derived from the simultaneous recordings of PERG and VEP.

It has been described that RCT, which does not represent a real “transit time” but an elecrophysiological index of the post-retinal function, is abnormal in OAG patients and it was suggested that glaucomatous visual field abnormalities can be ascribed to two sources of impairment: one at RGC-level and one involving the post-retinal structures [7]. In normal conditions, the post-retinal neural conduction is related to the formation and stabilization of synapsis between the RGC axons (afferent axons) and the LGN and the integrity of the neural connections between LGN (efferent axons) and the visual cortex [50,51]. Indeed, the lack or the reduction in bioelectrical activity from the RGCs to LGN induces loss or a dysfunction of LGN neural elements [50]. This condition was also observed in experimental glaucoma in deprivation of trophic factors and in the absence of axonal integrity [52,53,54].

Thus, in OAG, the observed delayed RCT [7] can be ascribed to different factors such as synaptic impairment between RGCs and LGN neural elements with consequent impairment of both magnocellular and parvocellular layers of the LGN [12,55], abnormal cortico-thalamic feedback [56], cortical atrophy [57], and demyelination within the optic radiations [58].

After treatment with Citicoline oral solution, in our OAG patients, a significant shortening of RCT was found. This suggests that this treatment may induce an improvement of the post-retinal neural conduction along both large and small axons.

Considering all neurophysiopathological mechanisms inducing abnormal RCT in OAG [7] to explain the observed improvement of the post-retinal neural conduction after treatment with Citicoline oral solution, several factors must be taken into consideration.

Firstly, we acknowledge that from the preclinical research studies, whose results cannot be directly translated in vivo in humans, Citicoline has neuromodulator effects due to its dopaminergic-like activity [18]. Based on this property, we can only hypothesize that the treatment with Citicoline oral solution provides an exogenous supplementation of neurotransmitters at the level of the RGC axons and LGN synapsis, likely inducing also in OAG human eyes a potentiation of these synapses. New synaptogenesis and synaptic pruning through a re-activation of basic synaptic mechanisms, althought not directly shown in this study, can be considered as an action of Citicoline on the visual system.

As a consequence, an improvement of the bioelectrical input from the RGC axons to the neuron of LGN and from those to the neurons reaching the visual cortex can be explained, eventually inducing a better bioelectrical activity of the visual cortex neurons, as measured electrophysiologically by the RCT shortening.

Although more robust imaging evidence is needed to make definitive conclusions, the interpretation of the neurophysiological changes under Citicoline treatment could be supported by our DTI data. In the Citicoline Group, we observed a difference in the mean values of the DTI parameters, although it did not reach statistical significance. This lack of significant changes may not indicate that the treatment does not induce significant microstructural changes but could be related to a statistical error of assessment. In fact, the sample size was calculated as a function of the primary outcome (VEP IT) and lacks statistical power for secondary morphological outcomes.

To the best of our knowledge, this is the first attempt to investigate brain microstructure after treatment with Citicoline in OAG patients

In animal models of stroke, Citicoline i.v. injections, or stereotactically delivered, yielded significantly smaller lesion volumes, verified recording DTI-MRI or MRI tracer, than controls [59,60].

In previous experimental studies in animals, inducing increased IOP and administering oral Citicoline, the DTI-MRI findings showed a reduced magnetization transfer ratio, a method for identifying myelin-related anomalies, at the level of the prechiasmatic optic nerves with respect to the animals with increased IOP without being treated with oral Citicoline [60].

Amongst the various MRI techniques, DTI can measure water diffusivity in tissues along the three main orthogonal diffusion axes, making it more sensitive to cellular and microstructural changes [61]. By computing fractional anisotropy, the overall white matter fibers anisotropy can be determined. In white matter, diffusion is preferentially enhanced in the direction of fiber orientation, indicating that reduced radial and/or increased axial diffusivity cause higher FA [62]. The mean diffusivity (MDi) parameter, comprising RD and AD, measures water diffusion and, indirectly, thermal changes and membrane contacts, and organelles drive water diffusion. Thus, cellular swelling or density may reduce MDi by blocking water diffusion. DTI can find major cerebral white matter anisotropic myelinated fiber tracts in gray matter nuclei like the LGN [63,64]. Only 20% of anisotropy is myelin. The amount of dendritic tree branching and crossing, local circuits, and axonal membranes also affects diffusive metrics in gray matter [65]. In our OAG patients administered with Citicoline oral solution, LGN and optic radiation exhibited bilaterally non-significant mean changes in FA and AD (increase) and in MDi and RD (reduction) at 12 months compared to the baseline. This trend of heightened anisotropy correlated with reduced mean diffusivity, and although not significant, it could suggest the contraction of neurons and glial cells and/or an enhancement of directional organization alongside cellular swelling.

Notably, animal models indicate that enhanced cell swelling may correlate with an augmented neural electrical response [65,66]. However, this interpretation should be handled with caution, considering the lack of significant changes observed after Citicoline treatment.

Notwithstanding, whatever the case may be, treatment with citicoline certainly induces a plastic change in the LGN and optic radiations.

It was interesting to observe that in the Citicoline Group, the increase in FA and AD and a reduction in MDi and RD tended to be associated (although not statically significantly) with the shortening of RCT and this suggests that in OAG patients, the treatment with Citicoline oral solution induces an enhancement of the post-retinal neural conduction that may be due to the associated morfolgical changes at the LGN and optic radiation levels.

Furthermore, there is an extensive literature regarding the positive effects of Citicoline to prevent the neurodegenerative process in brain diseases and, in particular, in acute stroke (see as a review Secades and Gareri [67]).

Second, it should also be considered that in experimental models, the treatment with Citicoline may induce axonal remyelination [68,69]. Since patterns of demyelination in the human post-mortem glaucomatous optic nerve have been observed [70], the possibility that Citicoline could induce axonal remyelination in our cohort of OAG patients cannot be entirely excluded.

### 4.4. Effect of Citicoline Oral Solution on Visual Field Defects

The OAG Group treated with Citicoline oral solution showed significant differences in HFA MD changes both at 6 and 12 months as compared to the Placebo Group. This is in agreement with the improvement of the VF detected by using Citicoline administred by eye drops [39,71]. We considered exclusively the differences in MD and PSD with respect to baseline merging data from the whole OAG Group, without subgrouping eyes based on the wide range of VF defects (ranging from −6 to −25 db of MD). A more refined statistical approach on the mean values of VF parameters in the Citicoline and Placebo Groups has not been applied since the present cohorts were not sufficiently numerous.

About the correlation between PERG 15′ A and HFA MD, by using Citicoline eye drops in OAG patients, a significant correlation between the improvement of RGC function and the amelioration of HFA MD was observed along a 4-month treatment period [39]. By contrast, in our cohort of OAG patients treated with Citicoline oral solution, no significant correlations between the increase in PERG amplitude and the increase in HFA MD were detected, leading us to believe that the improvement of the visual field is not exclusively related to the increased RGC function, and that other factors (see above, point 4.3) can be implicated.

On the other hand, improvement of the neural conduction along the large and small axons of the visual pathways (shortening of 60′ and 15′ VEP ITs) was significant linearly correlated with the reduction in the visual field defects (increase in HFA MD). This is in agreement with that observed in OAG patients treated with Citicoline eye drops [39].

Taken together, by considerening the above-mentioned lack of correlations between the increase in RGC function and the HFA MD, it is likely that when in OAG patients Citicoline is administred in oral solution, the changes in VF defects are not exclusively dependent on the enhancement of the RGC function but are related with the enhancement of the neural conduction along the whole visual pathways. In addition, the observed improvement of the VF, related to functional enhancement of the neural conduction along the visual pathways, should represent a beneficial effect for OAG patients’ vision after Citicoline oral solution treatment. Adeguated tests need to assess changes in real life, as previously reported by using Citicoline eye drops [22].

### 4.5. Study Limitations

Among the limitations of the present study, some critical points on the selection of our patients must be highlighted: the inclusion of OAG eyes with a wide visual field MD ranging from −6 to −25 dB forming a heterogeneous cohort from early to advanced OAG, the consideration of eyes only under beta-blocker monotherapy, which does not represent solely the treatment regimen of OAG, the exclusion of patients who underwent selective laser trabeculoplasty, the targeted IOP fixed <18 mmHg. Such elements induced us to select a small sample size of OAG eyes. Taken together, these limitations may have had an impact on the strength of correlations between ocular electrofunctional results and morphological post-retinal changes at the LGN level.

We also acknowledge the absence of OCT data regarding changes in GCL and RNFL thicknesses, given the present study exclusively focused on functional ophthalmological changes. In addition, the absence of collection of contrast sensitivity parameters and of patients’ self-reported outcomes limited the assessment of the impact of Citicoline in oral solution treatment on the patients’ quality of life, which has been previously described [22].

Additionally, we did not measure the ocular perfusion pressure, a crucial systemic vascular parameter, involved in the OAG pathogenesis, since our aim was mainly related to measurements of electrofunctional parameters along the visual pathways. Moreover, more evidence on DTI-MRI applied to glaucoma fields needs to be collected to generalize our neuroradiological evidence.

All that said, we acknowledge that while increasing the homogeneity of our sample, it has limited the external validity of our results.

## 5. Conclusions

In our cohort of OAG patients, the treatment with Citicoline oral solution induces a significant improvement of the function (neuroenhancement [18]) of RGCs. This effect can be ascribed to the above-mentioned mutifactorial mechanisms of action of Citicoline [18,20].

Other mechanisms of action, such as synaptic pruning, due to re-actication of synaptic plasticity induced by the neuromodulator effect of Citicoline, might explain the functional improvement of the visual pathways (in particular in post-retinal ones), with a consequent reduction in visual field defects.

Further studies using structural but also functional MRI techniques are required to assess localized morphological changes in the neural structures forming the visual pathways that can be likely modulated by Citicoline. To achieve this aim, a specifically powered study is warranted.

## Figures and Tables

**Table 1:** Descriptive statistics and effect size of Pattern Electroretinogram values observed in OAG patients treated with topic beta-blockers and Citicoline oral solution (Citicoline Group, 15 eyes) or treated with topic beta-blockers and Placebo (Placebo Group, 14 eyes). (**A**) Mean Values, 1 standard deviation, 95% confidence interval, and Cohen’s values; (**B**) One-way analysis of variance between Placebo and Citicoline Groups at baseline and each time point using Tukey’s method to correct for multiple comparisons; (**C**) One-way analysis of variance for Placebo and Citicoline Groups, respectively, at baseline and each time point (6 and 12 months) using Dunnett’s method to correct for multiple comparisons.

A	Citicoline Group	Placebo Group	Cohen’s d ^a^	B	ANOVA ^n^	Tukey ^o^	C	ANOVA ^n^ Placebo Group	Dunnett’s ^p^ Placebo Group	ANOVA ^n^ Citicoline Group	Dunnett’s ^p^ Citicoline Group
	Mean; 1 SD ^b^ (95% CI ^c^)	Mean, 1 SD ^b^ (95% CI ^c^)			F (5, 81) = *p* =	t = *p* =		F (2, 39) = *p* =	t = *p* =	F (2, 42) = *p* =	t = *p* =
60′ ^d^ IT ^e^ (ms) ^f^ BAS ^g^	61.153; 3.561 (59.181–63.125)	60.293; 1.862 (59.218–61.368)	0.30	60′ ^d^ IT ^e^ BAS ^g^	6.07 <0.001	−0.80 0.967					
60′ ^d^ IT ^e^ (ms) ^f^ 6 M ^h^	56.883; 3.628 (54.824–58.842)	61.421; 2.015 (60.258–62.585)	−1.53	60′ ^d^ IT ^e^ 6 M ^h^		4.24 0.001	60′ ^d^ IT ^e^ 6 M ^h^ vs. BAS ^g^	11.18 <0.001	1.48 0.250	9.94 <0.001	−3.34 0.003
60′ ^d^ IT ^e^ (ms) ^f^ 12 M ^h^	55.680; 3.433 (53.785–57.588)	63.821; 2.163 (62.573–65.070)	−2.81	60′ ^d^ IT ^e^ 12 M ^h^		5.39 <0.001	60′ ^d^ IT ^e^ 12 M ^h^ vs. BAS ^g^		4.63 <0.001		−4.23 <0.001
60′ ^b^ A ^i^ (µV) ^l^ BAS ^g^	1.633; 0.257 (1.491–1.775)	1.625; 0.404 (1.391–1.858)	0.02	60′ ^b^ A ^i^ BAS ^g^	17.18 <0.001	−0.05 1.000					
60′ ^b^ A ^i^ (µV) ^l^ 6 M ^h^	2.110; 0.234 (1.981–2.2340)	1.520; 0.422 (1.277–1.763)	1.75	60′ ^b^ A ^i^ 6 M ^h^		4.14 0.001	60′ ^b^ A ^g^ 6 M ^h^ vs. BAS ^g^	0.63 0.537	−0.66 0.733	23.96 <0.001	3.77 0.001
60′ ^b^ A ^i^ (µV) ^l^ 12 M ^h^	2.508; 0.490 (2.237–2.779)	1.448; 0.432 (1.199–1.697)	2.29	60′ ^b^ A ^i^ 12 M ^h^		7.44 <0.001	60′ ^b^ A ^g^ 12 M ^h^ vs. BAS ^g^		−1.12 0.435		6.91 <0.001
15′ ^m^ IT ^e^ (ms) ^f^ BAS ^g^	61.867; 3.502 (59.927–63.806)	62.143; 1.748 (61.134–63.152)	−0.01	15′ ^m^ IT ^e^ BAS ^g^	15.81 <0.001	−0.28 1.000					
15′ ^m^ IT ^e^ (ms) ^f^ 6 M ^h^	57.807; 3.161 (56.056–59.557)	63.557; 1.962 (62.424–64.690)	−0.22	15′ ^m^ IT ^e^ 6 M ^h^		−5.85 <0.001	15′ ^i^ IT ^e^ 6 M ^h^ vs. BAS ^g^	2.34 0.110	1.80 0.140	9.29 <0.001	−3.61 0.002
15′ ^m^ IT ^e^ (ms) ^f^ 12 M ^h^	57.533; 2.502 (56.148–58.919)	63.663; 2.452 (62.247–65.079)	−0.25	15′ ^m^ IT ^e^ 12 M ^h^		−6.23 <0.001	15′ ^i^ IT ^e^ 12 M ^h^ vs. BAS ^g^		1.94 0.107		−3.85 0.001
15′ ^m^ A ^i^ (µV) ^l^ BAS^g^	1.470; 0.427 (1.234–1.707)	1.606; 0.362 (1.397–1.815)	−0.51	15′ ^m^ A ^i^ BAS ^g^	10.49 <0.001	−0.98 0.924					
15′ ^m^ A ^i^ (µV) ^l^ 6 M ^h^	1.996; 0.380 (1.786–2.206)	1.518; 0.323 (1.332–1.704)	1.36	15′ ^m^ A ^i^ 6 M ^h^		3.44 0.012	15 ′^i^ A ^g^ 6 M ^h^ vs. BAS ^g^	1.15 0.327	−0.70 0.708	12.25 <0.001	3.51 0.002
15′ ^m^ A ^i^ (µV) ^l^ 12 M ^h^	2.185; 0.421 (1.951–2.418)	1.416; 0.309 (1.238–1.594)	2.08	15′ ^m^ A ^i^ 12 M ^h^		5.53 <0.001	15′ ^i^ A ^g^ 12 M ^h^ vs. BAS ^g^		−1.52 0.236		4.77 <0.001

^a^ Cohen’s d = Cohen’s d values; ^b^ 1 SD = one standard deviation; ^c^ CI = confidence interval; ^d^ 60′ = visual stimuli in which each check subtended 60 min of the visual arc, respectively; ^e^ IT = P50 Implicit time; ^f^ ms = milliseconds; ^g^ BAS = Baseline ^h^ M = Months; ^i^ A= P50–N95 Amplitude; ^l^ (µV) = microVolt; ^m^ 15′ = visual stimuli in which each check subtended 15 min of the visual arc; ^n^ ANOVA = One-way analysis of variance; ^o^ Tukey = Tukey comparisons method; ^p^ Dunnett’s = Dunnett’s comparisons method.

**Table 2:** Descriptive statistics and effect size of Visual Evoked Potentials values observed in OAG patients treated with topic beta-blockers and Citicoline oral solution (Citicoline Group, 15 eyes) or treated with topic beta-blockers and Placebo (Placebo Group, 14 eyes). (**A**) Mean Values, 1 standard deviation, 95% confidence interval, and Cohen’s values; (**B**) One-way analysis of variance between Placebo and Citicoline Groups at baseline and each time point using Tukey’s method to correct for multiple comparisons; (**C**) One-way analysis of variance for Placebo and Citicoline Groups, respectively, at baseline and each time point (6 and 12 months) using Dunnett’s method to correct for multiple comparisons.

A	Citicoline Group	Placebo Group	Cohen’s d ^a^	B	ANOVA ^n^	Tukey ^o^	C	ANOVA ^n^ Placebo Group	Dunnett’s ^p^ Placebo Group	ANOVA ^n^ Citicoline Group	Dunnett’s ^p^ Citicoline Group
	Mean; 1 SD ^b^ (95% CI ^c^)	Mean, 1 SD ^b^ (95% CI ^c^)			F (5, 81) = *p* =	t = *p* =		F (2, 39) = *p* =	t = *p* =	F (2, 42) = *p* =	t = *p* =
60′ ^d^ IT ^e^ (ms) ^f^ BAS ^g^	129.813; 6.719 (126.09–133.53)	127.021; 6.750 (123.12–130.92)	0.41	60′ ^d^ IT ^e^ BAS ^g^	10.23 <0.001	1.11 0.874					
60′ ^d^ IT ^e^ (ms) ^f^ 6 M ^h^	119.787; 5.794 (116.58–123.00)	130.643; 8.120 (125.95–135.33)	−1.31	60′ ^d^ IT ^e^ 6 M ^h^		−4.33 0.001	60′ ^d^ IT ^e^ 6 M ^h^ vs. BAS ^g^	1.58 0.219	1.29 0.337	14.91 <0.001	-4.53 <0.001
60′ ^d^ IT ^e^ (ms) ^f^ 12 M ^h^	118.944; 5.633 (115.82–122.06)	131.786; 7.266 (127.59–135.98)	−1.74	60′ ^d^ IT ^e^ 12 M ^h^		−5.13 <0.001	60′ ^d^ IT ^e^ 12 M ^h^ vs. BAS ^g^		1.70 0.169		−4.91 <0.001
60′ ^b^ A ^i^ (µV) ^l^ BAS ^g^	3.847; 1.120 (3.162–4.737)	3.950; 1.364 (3.16–4.738)	−0.07	60′ ^b^ A ^i^ BAS ^g^	7.35 <0.001	−0.21 1.000					
60′ ^b^ A^i^ (µV) ^l^ 6 M ^h^	5.180; 1.447 (4.378–5.982)	3.376; 1.439 (2.545–4.207)	1.25	60′ ^b^ A ^i^ 6 M ^h^		3.58 0.007	60′ ^b^ A ^g^ 6 M ^h^ vs. BAS ^g^	0.83 0.445	−1.09 0.452	7.48 0.002	2.78 0.015
60′ ^b^ A ^i^ (µV) ^l^ 12 M ^h^	5.633; 1.357 (4.88–6.884)	3.350; 1.382 (2.552–4.148)	1.65	60′ ^b^ A ^i^ 12 M ^h^		4.54 <0.001	60′ ^b^ A ^g^ 12 M ^h^ vs. BAS ^g^		−1.14 0.423		3.72 0.001
15′ ^m^ IT ^e^ (ms) ^f^ BAS ^g^	132.127; 8.571 (127.30–136.42)	129.143; 8.008 (124.52–133.77)	0.34	15′ ^m^ IT ^e^ BAS ^g^	7.60 <0.001	0.87 0.952					
15′ ^m^ IT ^e^ (ms) ^f^ 6 M ^h^	121.907; 7.111 (117.97–125.84)	131.786; 8.989 (126.60–136.98)	−1.08	15′ ^m^ IT ^e^ 6 M ^h^		−3.17 0.025	15′ ^i^ IT ^e^ 6 M ^h^ vs. BAS ^g^	0.69 0.505	0.78 0.656	11.80 <0.001	−3.50 0.002
15′ ^m^ IT ^e^ (ms) ^f^ 12 M ^h^	118.600; 7.962 (114.19–123.01)	133.071; 9.888 (127.37–138.78)	−1.44	15′ ^m^ IT ^e^ 12 M ^h^		−4.64 <0.001	15′ ^i^ IT ^e^ 12 M ^h^ vs. BAS ^g^		1.16 0.413		−4.67 <0.001
15′ ^m^ A ^i^ (µV) ^l^ BAS ^g^	3.313; 0.752 (2.897–3.730)	4.564; 1.536 (3.677–5.451)	−0.79	15′ ^m^ A ^i^ BAS^g^	4.54 0.001	−2.60 0.109					
15′ ^m^ A ^i^ (µV) ^l^ 6 M ^h^	4.673; 0.964 (4.142–5.212)	3.936; 1.486 (3.078–4.794)	0.48	15′ ^m^ A ^i^ 6 M ^h^		1.53 0.645	15′ ^i^ A ^g^ 6 M ^h^ vs. BAS ^g^	1.20 0.311	−1.08 0.455	14.16 <0.001	3.63 0.001
15′ ^m^ A ^i^ (µV) ^l^ 12 M ^h^	5.336; 1.294 (5.541–5.966)	3.693; 1.578 (2.781–4.604)	0.98	15′ ^m^ A ^i^ 12 M ^h^		3.24 0.021	15′ ^i^ A ^g^ 12 M ^h^ vs. BAS ^g^		−1.50 0.240		5.18 <0.001

^a^ Cohen’s d = Cohen’s d values; ^b^ 1 SD = one standard deviation; ^c^ CI = confidence interval; ^d^ 60′ = visual stimuli in which each check subtended 60 min of the visual arc, respectively; ^e^ IT = P100 Implicit time; ^f^ ms = milliseconds; ^g^ BAS = Baseline; ^h^ M = Months; ^i^ A = N75-P100 Amplitude; ^l^ (µV) = microVolt; ^m^ 15′ = visual stimuli in which each check subtended 15 min of the visual arc; ^n^ ANOVA = One-way analysis of variance; ^o^ Tukey = Tukey comparisons method; ^p^ Dunnett’s = Dunnett’s comparisons method.

**Table 3:** Descriptive statistics and effect size of Retinocortical Time (difference between VEP P100 and PERG P50 ITs) values observed in OAG patients treated with topic beta-blockers and Citicoline oral solution (Citicoline Group, 15 eyes) or treated with topic beta-blockers and Placebo (Placebo Group, 14 eyes). (**A**) Mean Values, 1 standard deviation, 95% confidence interval, and Cohen’s values; (**B**) One-way analysis of variance between Placebo and Citicoline Groups at baseline and each time point using Tukey’s method to correct for multiple comparisons; (**C**) One-way analysis of variance for Placebo and Citicoline Groups, respectively, at baseline and each time point (6 and 12 months) using Dunnett’s method to correct for multiple comparisons.

A	Citicoline Group	Placebo Group	Cohen’s d ^a^	B	ANOVA ^l^	Tukey ^m^	C	ANOVA ^l^ Placebo Group	Dunnett’s ^n^ Placebo Group	ANOVA ^l^ Citicoline Group	Dunnett’s ^n^ Citicoline Group
	Mean; 1 SD ^b^ (95% CI ^c^)	Mean, 1 SD ^b^ (95% CI ^c^)			F (5, 81) = *p* =	t = *p* =		F (2, 39) = *p* =	t = *p* =	F (2, 42) = *p* =	t = p =
60′ ^d^ RCT ^e^ (ms) ^f^ BAS ^g^	68.660; 4.674 (62.531–72.656)	66.729; 6.641 (62.889–70.558)	0.43	60′ ^d^ IT ^e^ BAS ^g^	3.58 0.006	1.30 0.780					
60′ ^d^ RCT ^e^ (ms) ^f^ 6 M ^h^	62.953; 4.473 (60.476–65.430)	69.221; 6.971 (65.197–73.247)	−0.88	60′ ^d^ IT ^e^ 6 M ^h^		−2.85 0.059	60′ ^d^ IT ^e^ 6 M ^h^ vs. BAS ^g^	0.48 0.625	0.98 0.524	8.46 0.001	−3.64 0.001
60′ ^d^ RCT ^e^ (ms) ^f^ 12 M ^h^	63.253; 4.918 (60.531–65.977)	67.694; 6.652 (64.124–71.805)	−0.65	60′ ^d^ IT ^e^ 12 M ^h^		−2.15 0.275	60′ ^d^ IT ^e^ 12 M ^h^ vs. BAS ^g^		0.48 0.845		−3.48 0.002
15′ ^i^ RCT ^e^ (ms) ^f^ BAS ^g^	70.260; 6.515 (66.652–73.87)	68.286; 7.436 (63.993–72.582)	0.26	15′ ^m^ IT ^e^ BAS ^g^	3.60 0.005	0.74 0.977					
15′ ^i^ RCT ^e^ (ms) ^f^ 6 M ^h^	64.100; 5.445 (61.084–67.116)	68.229; 7.769 (63.74–72.71)	−0.52	15′ ^m^ IT ^e^ 6 M ^h^		−1.54 0.640	15′ ^i^ IT ^e^ 6 M ^h^ vs. BAS ^g^	0.10; 0.909	−0.02 1.000	8.22 0.001	−2.67 0.020
15′ ^i^ RCT ^e^ (ms) ^f^ 12 M ^h^	61.067; 6.935 (57.226–64.907)	69.409; 8.898 (64.271–74.546)	−0.92	15′ ^m^ IT ^e^ 12 M ^h^		−3.11 0.030	15′ ^i^ IT ^e^ 12 M ^h^ vs. BAS ^g^		0.37 0.906		3.98 <0.001

^a^ Cohen’s d = Cohen’s d values; ^b^ 1SD = one standard deviation; ^c^ CI = confidence interval; ^d^ 60′ = visual stimuli in which each check subtended 60 min of the visual arc, respectively; ^e^ RCT = Retinocortical Time; ^f^ ms = milliseconds; ^g^ BAS = Baseline ^h^ M = Months; ^i^ 15′ = visual stimuli in which each check subtended 15 min of the visual arc; ^l^ ANOVA = One-way analysis of variance; ^m^ Tukey = Tukey comparisons method; ^n^ Dunnett’s = Dunnett’s comparisons method.

**Table 4:** Mean values and 1 standard deviation (±) of diffusion tensor imaging metrics values detected in the right and left nodes of visual pathways (optic radiations and LGN) detected at baseline and after 12 months of treatment in OAG patients treated with beta-blockers and Placebo (Placebo Group, 14 eyes) and in OAG patients treated with beta-blockers and Citicoline oral solution (Citicoline Group, 15 eyes).

Group	Time	Optic Radiations Right	Optic Radiations Left	LGN ^a^ Right	LGN ^a^ Left
Placebo	FA ^b^				
	Baseline	0.378 ± 0.032	0.355 ± 0.019	0.315 ± 0.094	0.416 ± 0.114
	12 months	0.375 ± 0.027	0.351 ± 0.013	0.311 ± 0.093	0.419 ± 0.089
	MDi ^c^ (mm^2^/s)				
	Baseline	8.762 × 10^4^ ± 7.520 × 10^5^	9.399 × 10^4^ ± 8.168 × 10^5^	1.198 × 10^3^ ± 2.613 × 10^4^	1.010 × 10^3^ ± 2.608 × 10^4^
	12 months	8.858 × 10^4^ ± 7.373 × 10^5^	9.268 × 10^4^ ± 5.850 × 10^5^	1.219 × 10^3^ ± 3.287 × 10^4^	1.884 × 10^4^ ± 1.492 × 10^4^
	AD ^d^ (mm^2^/s)				
	Baseline	1.234 × 10^3^ ± 9.979 × 10^5^	1.287 × 10^3^ ± 1.031 × 10^4^	1.544 × 10^3^ ± 2.878 × 10^4^	1.406 × 10^3^ ± 2.356 × 10^4^
	12 months	1.245 × 10^3^ ± 9.929 × 10^5^	1.267 × 10^3^ ± 6.736 × 10^5^	1.568 × 10^3^ ± 3.626 × 10^4^	1.263 × 10^3^ ± 1.563 × 10^4^
	RD ^e^ (mm^2^/s)				
	Baseline	7.232 × 10^4^ ± 1.243 × 10^4^	7.898 × 10^4^ ± 1.255 × 10^4^	1.053 × 10^3^ ± 3.102 × 10^4^	8.368 × 10^4^ ± 3.220 × 10^4^
	12 months	7.063 × 10^4^ ± 6.457 × 10^5^	7.564 × 10^4^ ± 5.569 × 10^5^	1.045 × 10^3^ ± 3.199 × 10^4^	7.015 × 10^4^ ± 1.610 × 10^4^
Citicoline	FA ^b^				
	Baseline	0.391 ± 0.029	0.341 ± 0.019	0.324 ± 0.102	0.405 ± 0.151
	12 months	0.414 ± 0.033	0.365 ± 0.019	0.348 ± 0092	0.423 ± 0.078
	MDi ^c^ (mm^2^/s)				
	Baseline	8.795 × 10^4^ ± 3.778 × 10^5^	9.542 × 10^4^ ± 5.798 × 10^5^	1.375 × 10^3^ ± 3.524 × 10^4^	1.154 × 10^3^ ± 4.612 × 10^4^
	12 months	8.582 × 10^4^ ± 3.478 × 10^5^	9.365 × 10^4^ ± 6.517 × 10^5^	1.220 × 10^3^ ± 4.326 × 10^4^	1.018 × 10^3^ ± 3.801 × 10^4^
	AD ^d^ (mm^2^/s)				
	Baseline	1.269 × 10^3^ ± 3.155 × 10^5^	1.269 × 10^3^ ± 7.668 × 10^5^	1.514 × 10^3^ ± 3.538 × 10^4^	1.525 × 10^3^ ± 4.362 × 10^4^
	12 months	1.424 × 10^3^ ± 3.772 × 10^5^	1.469 × 10^3^ ± 8.645 × 10^5^	1.779 × 10^3^ ± 4.471 × 10^4^	1.605 × 10^3^ ± 4.069 × 10^4^
	RD ^e^ (mm^2^/s)				
	Baseline	6.650 × 10^4^ ± 4.388 × 10^5^	7.669 × 10^4^ ± 5.073 × 10^5^	1.005 × 10^3^ ± 3.625 × 10^4^	8.938 × 10^4^ ± 4.838 × 10^4^
	12 months	6.454 × 10^4^ ± 4.179 × 10^5^	7.505 × 10^4^ ± 5.683 × 10^5^	9.400 × 10^4^ ± 4.289 × 10^4^	8.252 × 10^4^ ± 3.736 × 10^4^

^a^ LGN = lateral geniculate nucleus; ^b^ FA = fractional anisotropy; ^c^ MDi = mean diffusivity; ^d^ AD = axial diffusivity, and ^e^ RD = radial diffusivity.

## Data Availability

Data supporting reported results are available upon request to the corresponding author.

## Supplementary Materials

The following supporting information can be downloaded at https://www.mdpi.com/article/10.3390/jcm15010223/s1, Table S1: Changes in PERG (P50 implicit times and P50–N95 amplitudes) and VEP (P100 implicit times and 75-P100 amplitudes) parameters and RCT values after 6 and 12 months of treatment with respect to the baseline condition observed in OAG patients treated with topic beta-blockers and Placebo (Placebo Group, 14 eyes) and in OAG patients treated with topic beta-blockers and Citicoline oral solution (Citicoline Group, 15 eyes). Table S2: Descriptive statistics and effect size of individual changes (6 months minus baseline and 12 months minus baseline) of Pattern Electroretinogram (PERG) and Visual Evoked Potentials (VEPs) parameters and Retinocortical Time (RCT, difference between VEP P100 and PERG P50 ITs) values detected in OAG patients treated with beta-blockers and Placebo (Placebo Group, 14 eyes) and in OAG patients treated with topic beta-blockers and Citicoline oral solution (Citicoline Group, 15 eyes). (A) Mean Values, 1 standard deviation, 95% confidence interval, and Cohen’s values; (B) One-way analysis of variance between Placebo and Citicoline Groups at baseline and each time point using Tukey’s method to correct for multiple comparisons.

## Abbreviations

AD	axial diffusivity
ANOVA	one-way analysis of variance
AP	anterior–posterior
BCVA	best-corrected visual acuity
CLs	confidence limits
DTI	Diffusion Tensor Imaging
DTIFIT	Diffusion tensor imaging fit
ETDRS	Early Treatment Diabetic Retinopathy Study
FA	fractional anisotropy
ff-ERG	full-field electroretinogram
FOV	field of view
FWE	free water elimination
GCL	ganglion cells layer
GCL-T	ganglion cells layer thickness
HFA	Humphrey Field Analyzer
IN	inferior-nasal
INL	inner nuclear layer
IOP	intraocular pressure
IPL	inner plexiform layer
ISCEV	Internation Society for Clinical Electrophysiology of Vision
IT	inferior-temporal
LGN	lateral geniculate nucleus
logMAR	logarithm of the Minimum Angle of Resolution
MD	mean deviation
MDi	mean diffusivity
mfERG	multifocal electroretinogram
mfPhNR	multifocal Photopic Negative Response
MS-ON	multiple sclerosis-optic neuritis
MRI	magnetic resonance imaging
N	number of eyes of each group
OAG	open-angle glaucoma
OCT	optical coherence tomography
OHT	ocular hypertension
PA	posterior–anterior
PERG	pattern electroretinogram
PhNR	Photopic Negative Response
PSD	Pattern Standard Deviation
RCT	Retinocortical Time
RAD	response amplitude density
RD	radial diffusivity
RGCs	retinal ganglion cells
RNFL	retinal nerve fiber layer
ROI	regions of interest
SD	one standard deviation of the mean
SD-OCT	spectral domain-optical coherence tomography
SN	superior-nasal
ST	superior-temporal
SNR	Signal to noise ratio
TBSS	Tract-Based Spatial Statistics
TE	echo time
TR	Repetition time
VEP	visual evoked potentials
VF	visual field

## References

[1] Quigley H.A. (2011). Glaucoma. Lancet.

[2] American Academy of Ophthalmology (AAO) (2010). Primary Angle Closure Glaucoma. Preferred Practice Pattern Guidelines.

[3] Quigley H.A., McKinnon S.J., Zack D.J., Pease M.E., Kerrigan-Baumrind L.A., Kerrigan D.F., Mitchell R.S. (2000). Retrograde axonal transport of BDNF in retinal ganglion cells is blocked by acute IOP elevation in rats. Investig. Ophthalmol. Vis. Sci..

[4] Lipton S.A., Rosenberg P.A. (1994). Excitatory amino acids as a final common pathway for neurologic disorders. N. Engl. J. Med..

[5] Guo L., Salt T.E., Maass A., Luong V., Moss S.E., Fitzke F.W., Cordeiro M.F. (2006). Assessment of neuroprotective effects of glutamate modulation on glaucoma-related retinal ganglion cell apoptosis in vivo. Investig. Ophthalmol. Vis. Sci..

[6] Burgoyne C.F., Downs J.C., Bellezza A.J., Suh J.K., Hart R.T. (2005). The optic nerve head as a biomechanical structure: A new paradigm for understanding the role of IOP-related stress and strain in the pathophysiology of glaucomatous optic nerve head damage. Prog. Retin. Eye Res..

[7] Parisi V., Tanga L., Roberti G., Barbano L., Carnevale C., Manni G., Oddone F. (2021). Neural conduction along post-retinal visual pathways in glaucoma. Front. Aging Neurosci..

[8] Furlanetto R.L., Teixeira S.H., Gracitelli C.P.B., Lottenberg C.L., Emori F., Michelan M., Amaro E., Paranhos A. (2018). Structural and functional analyses of the optic nerve and lateral geniculate nucleus in glaucoma. PLoS ONE.

[9] Schmidt M.A., Knott M., Heidemann R., Michelson G., Kober T., Dörfler A., Engelhorn T. (2018). Investigation of lateral geniculate nucleus volume and diffusion tensor imaging in patients with normal tension glaucoma using 7 tesla magnetic resonance imaging. PLoS ONE.

[10] Aksoy D.Ö., Umurhan Akkan J.C., Alkan A., Aralaşmak A., Otçu Temur H., Yurtsever İ. (2019). Magnetic resonance spectroscopy features of the visual pathways in patients with glaucoma. Clin. Neuroradiol..

[11] Wang Y., Wang X., Zhou J., Qiu J., Yan T., Xie Y., Li L., Lu W. (2020). Brain morphological alterations of cerebral cortex and subcortical nuclei in high-tension glaucoma brain and its associations with intraocular pressure. Neuroradiology.

[12] Yucel Y.H., Zhang Q., Weinreb R.N., Kaufman P.L., Gupta N. (2003). Effects of retinal ganglion cell loss on magno-, parvo-, koniocellular pathways in the lateral geniculate nucleus and visual cortex in glaucoma. Prog. Retin. Eye Res..

[13] Gracitelli C.P.B., Duque-Chica G.L., Sanches L.G., Moura A.L., Nagy B.V., Teixeira S.H., Amaro E., Ventura D.F., Paranhos A. (2020). Structural analysis of glaucoma brain and its association with ocular parameters. J. Glaucoma.

[14] Zhang Q., Shu Y., Li X., Xiong C., Li P., Pang Y., Ye W., Yang L., Zeng X., Zhang X. (2019). Resting-state functional magnetic resonance study of primary open-angle glaucoma based on voxelwise brain network degree centrality. Neurosci. Lett..

[15] Cio F.D., Garaci F., Minosse S., Passamonti L., Martucci A., Lanzafame S., Giuliano F.D., Picchi E., Mancino R., Guerrisi M. (2020). Disruption of structural brain networks in Primary Open Angle Glaucoma. Annu. Int. Conf. IEEE Eng. Med. Biol. Soc..

[16] Chang E.E., Goldberg J.L. (2012). Glaucoma 2.0: Neuroprotection, neuroregeneration, neuroenhancement. Ophthalmology.

[17] Bou Ghanem G.O., Wareham L.K., Calkins D.J. (2024). Addressing neurodegeneration in glaucoma: Mechanisms, challenges, and treatments. Prog. Retin. Eye Res..

[18] Faiq M.A., Wollstein G., Schuman J.S., Chan K.C. (2019). Cholinergic nervous system and glaucoma: From basic science to clinical applications. Prog. Retin. Eye Res..

[19] Sbardella D., Coletta A., Tundo G.R., Ahmed I.M.M., Bellia F., Oddone F., Manni G., Coletta M. (2020). Structural and functional evidence for citicoline binding and modulation of 20S proteasome activity: Novel insights into its pro-proteostatic effect. Biochem. Pharmacol..

[20] Oddone F., Rossetti L., Parravano M., Sbardella D., Coletta M., Ziccardi L., Roberti G., Carnevale C., Romano D., Manni G. (2021). Citicoline in Ophthalmological Neurodegenerative Disease: A Comprehensive Review. Pharmaceuticals.

[21] Rejdak R., Toczolowski J., Krukowski J., Kaminski M., Rejdak K., Stelmasiak Z., Grieb P. (2003). Oral citicoline treatment improves visual pathway function in glaucoma. Med. Sci. Monit..

[22] Rossetti L., Goni F., Montesano G., Stalmans I., Topouzis F., Romano D., Galantin E., Delgado-Gonzales N., Giammaria S., Coco G. (2023). The effect of citicoline oral solution on quality of life in patients with glaucoma: The results of an international, multicenter, randomized, placebo-controlled cross-over trial. Graefes Arch. Clin. Exp. Ophthalmol..

[23] Yohannan J., Wang J., Brown J., Chauhan B.C., Boland M.V., Friedman D.S., Ramulu P.Y. (2017). Evidence-based Criteria for Assessment of Visual Field Reliability. Ophthalmology.

[24] Falsini B., Colotto A., Porciatti V., Porrello G. (1992). Follow-up study with pattern ERG in ocular hypertension and glaucoma patients under timolol maleate treatment. Clin. Vision Sci..

[25] Ventura L., Porciatti V. (2005). Restoration of retinal ganglion cell function in early glaucoma after intraocular pressure reduction. Ophthalmology.

[26] Nesher R., Trick G.L., Kass M.A., Gordon M.O. (1990). Steady-state pattern electroretinogram following long term unilateral administration of timolol to ocular hypertensive subjects. Doc. Ophthalmol..

[27] Colotto A., Salgarello T., Giudiceandrea A., De Luca L.A., Coppè A., Buzzonetti L., Falsini B. (1995). Pattern electroretinogram in treated ocular hypertension: A cross-sectional study after timolol maleate therapy. Ophthalmic Res..

[28] Odom J.V., Bach M., Brigell M., Holder G.E., McCulloch D.L., Mizota A., Tormene A.P. (2016). ISCEV standard for clinical visual evoked potentials–(2016 update). Doc. Ophthalmol..

[29] Smith S.M., Jenkinson M., Woolrich M.W., Beckmann C.F., Behrens T.E., Johansen-Berg H., Bannister P.R., De Luca M., Drobnjak I., Flitney D.E. (2004). Advances in functional and structural MR image analysis and implementation as FSL. NeuroImage.

[30] Woolrich M.W., Jbabdi S., Patenaude B., Chappell M., Makni S., Behrens T., Beckmann C., Jenkinson M., Smith S.M. (2009). Bayesian analysis of neuroimaging data in FSL. NeuroImage.

[31] Jenkinson M., Beckmann C.F., Behrens T.E.J., Woolrich M.W., Smith S.M. (2012). FSL. NeuroImage.

[32] Andersson J.L., Skare S., Ashburner J. (2003). How to correct susceptibility distortions in spin-echo echo-planar images: Application to diffusion tensor imaging. NeuroImage.

[33] Smith S.M. (2002). Fast robust automated brain extraction. Hum. Brain Mapp..

[34] Bastiani M., Cottaar M., Fitzgibbon S.P., Suri S., Alfaro-Almagro F., Sotiropoulos S.N., Jbabdi S., Andersson J.L. (2019). Automated quality control for within and between studies diffusion MRI data using a non-parametric framework for movement and distortion correction. NeuroImage.

[35] Kastner S., O’Connor D.H., Fukui M.M., Fehd H.M., Herwig U., Pinsk M.A. (2004). Functional Imaging of the Human Lateral Geniculate Nucleus and Pulvinar. J. Neurophysiol..

[36] Jenkinson M., Smith S. (2001). A global optimisation method for robust affine registration of brain images. Med. Image Anal..

[37] Jenkinson M., Bannister P., Brady M., Smith S. (2002). Improved optimization for the robust and accurate linear registration and motion correction of brain images. NeuroImage.

[38] Smith S.M., Jenkinson M., Johansen-Berg H., Rueckert D., Nichols T.E., Mackay C.E., Watkins K.E., Ciccarelli O., Cader M.Z., Matthews P.M. (2006). Tract-based spatial statistics: Voxelwise analysis of multi-subject diffusion data. Neuroimage.

[39] Parisi V., Centofanti M., Ziccardi L., Tanga L., Michelessi M., Roberti G., Manni G. (2015). Treatment with Citicoline eye drops enhances retinal function and neural conduction along the visual pathways in open angle glaucoma. Graefes Arch. Clin. Exp. Ophthalmol..

[40] Thompson D.A., Bach M., McAnany J.J., Šuštar Habjan M., Viswanathan S., Robson A.G. (2024). ISCEV standard for clinical pattern electroretinography (2024 update). Doc. Ophthalmol..

[41] Parisi V., Miglior S., Manni G., Centofanti M., Bucci M.G. (2006). Clinical ability of pattern electroretinograms and visual evoked potentials in detecting visual dysfunction in ocular hypertension and glaucoma. Ophthalmology.

[42] Agut J., Font E., Sacrist A., Ortiz J.A. (1983). Bioavailability of Methyl-14C CDP-Choline by Oral Route. Arzneimittelforschung.

[43] Roda A., Fini A., Grigolo B., Scapini G. (1983). Routes of administration and serum levels of [Methyl-14C]-Cytidine-Diphosphocholine. Curr. Ther. Res..

[44] Parisi V., Manni G., Colacino G., Bucci M.G. (1999). Cytidine-5′-diphosphocholine (citicoline) improves retinal and cortical responses in patients with glaucoma. Ophthalmology.

[45] Parisi V., Coppola G., Centofanti M., Oddone F., Angrisani A.M., Ziccardi L., Ricci B., Quaranta L., Manni G. (2008). Evidence of the neuroprotective role of citicoline in glaucoma patients. Prog. Brain Res..

[46] Oshitari T., Fujimoto N., Adachi-Usami E. (2002). Citicoline has a protective effect on damaged retinal ganglion cells in mouse culture retina. Neuroreport.

[47] Oshitari T., Yoshida-Hata N., Yamamoto S. (2010). Effect of neurotrophic factors on neuronal apoptosis and neurite regeneration in cultured rat retinas exposed to high glucose. Brain Res..

[48] Matteucci A., Varano M., Gaddini L., Mallozzi C., Villa M., Pricci F., Malchiodi-Albedi F. (2014). Neuroprotective effects of citicoline in in vitro models of retinal neurodegeneration. Int. J. Mol. Sci..

[49] Prinz J., Prokosch V., Liu H., Walter P., Fuest M., Migliorini F. (2023). Efficacy of citicoline as a supplement in glaucoma patients: A systematic review. PLoS ONE.

[50] Parisi V., Scarale M.E., Balducci N., Fresina M., Campos E.C. (2010). Electrophysiological detection of delayed postretinal neural conduction in human amblyopia. Investig. Ophthalmol. Vis. Sci..

[51] Hubel D.H., Wiesel T.N. (1965). Binocular interaction in striate cortex of kittens reared with artificial squint. J. Neurophysiol..

[52] Pease M.E., McKinnon S.J., Quigley H.A., Kerrigan-Baumrind L.A., Zack D.J. (2000). Obstructed axonal transport of BDNF and its receptor TrkB in experimental glaucoma. Investig. Ophthalmol. Vis. Sci..

[53] Soto I., Oglesby E., Buckingham B.P., Son J.L., Roberson E.D., Steele M.R., Inman D.M., Vetter M.L., Horner P.J., Marsh-Armstrong N. (2008). Retinal ganglion cells downregulate gene expression and lose their axons within the optic nerve head in a mouse glaucoma model. J. Neurosci..

[54] Crish S.D., Sappington R.M., Inman D.M., Horner P.J., Calkins D.J. (2010). Distal axonopathy with structural persistence in glaucomatous neurodegeneration. Proc. Natl. Acad. Sci. USA.

[55] Weber A.J., Chen H., Hubbard W.C., Kaufman P.L. (2000). Experimental glaucoma and cell size, density, and number in the primate lateral geniculate nucleus. Investig. Ophthalmol. Vis. Sci..

[56] Baroncelli L., Lunghi C. (2021). Neuroplasticity of the visual cortex: In sickness and in health. Exp. Neurol..

[57] Gupta N., Krishnadev N., Hamstra S.J., Yücel Y.H. (2006). Depth perception deficits in glaucoma suspects. Br. J. Ophthalmol..

[58] Kaushik M., Stuart L., Graham S.L., Wang C., Klistorner A. (2014). A topographical relationship between visual field defects and optic radiation changes in glaucoma. Investig. Ophthalmol. Vis. Sci..

[59] Ramos-Cabrer P., Agulla J., Argibay B., Pérez-Mato M., Castillo J. (2011). Serial MRI study of the enhanced therapeutic effects of liposome-encapsulated citicoline in cerebral ischemia. Int. J. Pharm..

[60] Xu F., Han H., Yan J., Chen H., He Q., Xu W., Zhu N., Zhang H., Zhou F., Lee K. (2011). Greatly improved neuroprotective efficiency of citicoline by stereotactic delivery in treatment of ischemic injury. Drug Deliv..

[61] Van der Merwe Y., Murphy M.C., Sims J.R., Faiq M.A., Yang X.L., Ho L.C., Conner I.P., Yu Y., Leung C.K., Wollstein G. (2021). Citicoline Modulates Glaucomatous Neurodegeneration Through Intraocular Pressure-Independent Control. Neurotherapeutics.

[62] Basser P., Mattiello J., LeBihan D. (1994). MR diffusion tensor spectroscopy and imaging. Biophys. J..

[63] Alexander A., Lee J., Lazar M., Field A. (2007). Diffusion tensor imaging of the brain. Neurotherapeutics.

[64] Sedrak M., Gorgulho A., Frew A., Behnke E., DeSalles A., Pouratian N. (2011). Diffusion tensor imaging and colored fractional anisotropy mapping of the ventralis intermedius nucleus of the thalamus. Neurosurgery.

[65] Mang S., Busza A., Reiterer S., Grodd W., Klose A. (2012). Thalamus segmentation based on the local diffusion direction: A group study. Magn. Reson. Med..

[66] Beaulieu C. (2002). The basis of anisotropic water diffusion in the nervous system-a technical review. NMR Biomed..

[67] Secades J.J., Gareri P. (2022). Citicoline: Pharmacological and clinical review, 2022 update. Rev. Neurol..

[68] Skripuletz T., Manzel A., Gropengießer K., Schäfer N., Gudi V., Singh V., Salinas Tejedor L., Jörg S., Hammer A., Voss E. (2015). Pivotal role of choline metabolites in remyelination. Brain.

[69] Gudi V., Schäfer N., Gingele S., Stangel M., Skripuletz T. (2021). Regenerative Effects of CDP-Choline: A Dose-Dependent Study in the Toxic Cuprizone Model of De-and Remyelination. Pharmaceuticals.

[70] Parrilla G.E., Salkar A., Wall R.V., Gupta V., Graham S.L., You Y. (2024). Glaucoma, More than Meets the Eye: Patterns of Demyelination Revealed in Human Postmortem Glaucomatous Optic Nerve. Aging Dis..

[71] Rossetti L., Iester M., Tranchina L., Ottobelli L., Coco G., Calcatelli E., Ancona C., Cirafici P., Manni G. (2020). Can Treatment with Citicoline Eyedrops Reduce Progression in Glaucoma? The Results of a Randomized Placebo-controlled Clinical Trial. J. Glaucoma.

