# Supplementary material for: Citicoline Oral Solution Induces Functional Enhancement and Synaptic Plasticity in Patients with Open-Angle Glaucoma
Source: J Clin Med. 2025 Dec 27;15(1):223. doi: 10.3390/jcm15010223 (PMC12786903; doi:10.3390/jcm15010223)
Supplement: Supplementary file 1 [file jcm-15-00223-s001.zip › jcm-4047785-supplementary.pdf]

SUPPLEMENTARY MATERIALS

Table S1 Changes of PERG (P50 implicit times and P50-N95 amplitudes) and VEP (P100 implicit times and 75-P100 amplitudes) parameters and RCT values after 6 and 12 months of treatment with respect to the baseline condition observed in OAG patients treated with topic beta-blockers and placebo (Placebo Group, 14 eyes) and in OAG patients treated with topic beta-blockers and Citicoline oral solution (Citicoline Group, 15 eyes).

	**Placebo Group (14 eyes)**						**Citicoline Group (15 eyes)**					
	**Unmodified** [a]		**Improvement** [b]		**worsening** [c]		**Unmodified** [a]		**Improvement** [b]		**worsening** [c]	
	**N** [d]	**%**	**N** [d]	**%**	**N** [d]	**%**	**N** [d]	**%**	**N** [d]	**%**	**N** [d]	**%**
Difference 6 months minus baseline												
60′ [e] PERG P50 IT [f]	13	92.86	0	0.00	1	7.14	4	26.67	11	73.33	0	0.00
60′ [e] PERG P50-N95 A [g]	13	92.86	0	0.00	1	7.14	4	26.67	11	73.33	0	0.00
15′ [h] PERG P50 IT [f]	13	92.86	0	0.00	1	7.14	1	6.67	14	93.33	0	0.00
15′ [h] PERG P50-N95 A [g]	12	85.71	0	0.00	2	14.29	3	20.00	12	80.00	0	0.00
60′ [e] VEP P100 IT [f]	10	71.43	0	0.00	4	28.57	3	20.00	12	80.00	0	0.00
60′ [e] VEP N75-P100 A [g]	11	78.57	0	0.00	3	21.43	7	46.67	8	53.33	0	0.00
15′ [h] VEP P100 IT [f]	11	78.57	0	0.00	3	21.43	3	20.00	12	80.00	0	0.00
15′ [h] VEP N75-P100 A [g]	11	78.57	0	0.00	3	21.43	5	33.33	10	66.67	0	0.00
60′ [e] RCT	10	71.43	0	0.00	4	28.57	2	13.33	11	73.33	2	13.33
15′ [f] RCT	6	42.86	4	28.57	4	28.57	2	13.33	12	80.00	1	6.67
Difference 12 months minus baseline												
60′ [e] PERG P50 IT [f]	13	92.86	1	7.14	0	0.00	1	6.67	14	93.33	0	0.00
60′ [e] PERG P50-N95 A [g]	11	78.57	0	0.00	3	21.43	1	6.67	14	93.33	0	0.00
15′ [h] PERG P50 IT [f]	11	78.57	0	0.00	3	21.43	2	13.33	13	86.67	0	0.00
15′ [h] PERG P50-N95 A [g]	11	78.57	0	0.00	3	21.43	0	0.00	15	100.00	0	0.00
60′ [e] VEP P100 IT [f]	7	50.00	0	0.00	7	50.00	3	20.00	12	80.00	0	0.00
60′ [e] VEP N75-P100 A [g]	12	85.71	0	0.00	2	14.29	2	13.33	13	86.67	0	0.00
15′ [h] VEP P100 IT [f]	11	78.57	0	0.00	3	21.43	1	6.67	14	93.33	0	0.00
15′ [h] VEP N75-P100 A [g]	12	85.71	0	0.00	2	14.29	1	6.67	14	93.33	0	0.00
60′ [e] RCT	7	50.00	0	0.00	7	50.00	2	13.33	11	73.33	2	13.33
15′ [h] RCT	10	71.43	2	14.29	2	14.29	4	26.67	11	73.33	0	0.00

[a] Unmodified = changes within the 95% confidence test-retest limit; [b] improvement = increase in amplitudes and shortening in implicit times that exceeded the 95% confidence test-retest limit; [c] worsening = reduction in amplitudes and increase in implicit times that exceeded the 95% confidence test-retest limit; [d] N= number of eyes; [e] 60′ = visual stimuli in which each check subtended 60 minutes of the visual arc; [f] IT= implicit time; [g] A= amplitude; [h] 15′ = visual stimuli in which each check subtended 15 minutes of the visual arc.

Table S2. Descriptive statistics and effect size of individual changes (6 months minus baseline and 12 months minus baseline) of Pattern Electtroretinogram (PERG) and Visual Evoked Potentials (VEP) parameters and Retinocortical time (RCT, difference between VEP P100 and PERG P50 ITs) values detected in OAG patients treated with topic beta-blockers and placebo (Placebo Group, 14 eyes) and in OAG patients treated with topic beta-blockers and Citicoline oral solution (Citicoline Group, 15 eyes). **A**: Mean Values, 1 standard deviation, 95% confidence interval and Choen's values; **B:** One-way analysis of variance between Placebo and Citicoline Groups at baseline each time point using Tukey's method to correct for multiple comparisons.

**A**	**Citicoline Group**	**Placebo Group**	**Cohen's d** [a]	**B**	**ANOVA** [n]	**Tukey** [o]
	**Mean; 1SD**[b]<br>**(95% CI**[c]**)**	**mean, 1SD**[b]<br>**(95% CI**[c]**)**			**F (5, 81) =**<br>**p =**	**t =**<br>**p =**
60′[d] PERG P50 IT[e] (logms)[f]<br>6 M[g] minus BAS[h]	-0.032; 0.014<br>(-0.04- -0.024)	0.008; 0.012<br>(0.001–0.0147)	-3.06	60′[d] PERG P50 IT[e]<br>6 M[g] minus BAS[h]	86.28<br><0.001	-8.37<br><0.001
60′[d] PERG P50 IT[e] (logms)[f]<br>12 M[g] minus BAS[h]	0.025; 0.012<br>(-0.048- -0.033)	-0.041; 0.014<br>(0.018–0.031)	5.08	60′[d] PERG P50 IT[e]<br>12 M[g] minus BAS[h]		-13.70<br><0.001
60′[d] PERG P50-N95 A[i] (logμV)[l]<br>6 M[g] minus BAS[h]	0.114; 0.057<br>(0.082– 0.146)	-0.032; 0.039<br>(-0.054- -0.009)	2.97	60′[d] PERG P50-N95 A[i]<br>6 M[g] minus BAS[h]	52.45<br><0.001	6.47<br><0.001
60′[d] PERG P50-N95 A[i] (logμV)[l]<br>12 M[g] minus BAS[h]	0.184; 0.088<br>0.135–0.233	-0.056; 0.043<br>(-0.080- -0.031)	3.39	60′[d] PERG P50-N95 A[i]<br>12 M[g] minus BAS[h]		10.63<br><0.001
60′[d] VEP P100 IT[e] (logms)[f]<br>6 M[g] minus BAS[h]	-0.029; 0.0185<br>(-0.039- -0.019)	0.010; 0.006<br>(0.006–0.013)	-2.79	60′[d] VEP P100 IT[e]<br>6 M[g] minus BAS[h]	37.70<br><0.001	-7.29<br><0.001
60′[d] VEP P100 IT[e] (logms)[f]<br>12 M[g] minus BAS[h]	-0.031; 0.0187<br>(-0.041- -0.021)	0.010; 0.011<br>(0.004–0.016)	-2.65	60′[d] VEP P100 IT[e]<br>12 M[g] minus BAS[h]		-7.74<br><0.001
60′[d] VEP N75-P100 A[i] (logμV)[l]<br>6 M[g] minus BAS[h]	0.145; 0.068<br>(0.107-0.182)	-0.023; 0.040<br>(-0.046- -0.0003)	2.98	60′[d] VEP N75-P100 A[i]<br>6 M[g] minus BAS[h]	50.51<br><0.001	7.10<br><0.001
60′[d] VEP N75-P100 A[i] (logμV)[l]<br>12 M[g] minus BAS[h]	0.184; 0.083<br>(0.138-0.23)	-0.054; 0.054<br>(-0.085- -0.023)	3.37	60′[d] VEP N75-P100 A[i]<br>12 M[g] minus BAS[h]		-10.05<br><0.001
60′[d] RCT (logms)[f]<br>6 M[g] minus BAS[h]	-0.035; 0.021<br>(-0.046- -0.0234)	0.012; 0.007<br>(0.008–0.016)	-2.96	60′[d] RCT<br>6 M[g] minus BAS[h]	46.57<br><0.001	7.76<br><0.001
60′[d] RCT (logms)[f]<br>12 M[g] minus BAS[h]	-0.038; 0.022<br>(-0.050- -0.026)	0.016; 0.009<br>(0.011–0.021)	-3.17	60′[d] RCT<br>12 M[g] minus BAS[h]		-8.92<br><0.001
15′[m] PERG P50 IT[e] (logms)[f]<br>6 M[g] minus BAS[h]	0.130; 0.071<br>(0.091- 0.169)	-0.075; 0.072<br>(-0.117- -0.033)	2.87	15′[m] PERG P50 IT[e]<br>6 M[g] minus BAS[h]	45.56<br><0.001	7.43<br><0.001
15′[m] PERG P50 IT[e] (logms)[f]<br>12 M[g] minus BAS[h]	0.171; 0.067<br>(0.134-0.208)	-0.077; 0.087<br>(-0.127- -0.027)	3.21	15′[m] PERG P50 IT[e]<br>12 M[g] minus BAS[h]		8.96<br><0.001
15′[m] PERG P50-N95 A[i] (logμV)[l]<br>6 M[g] minus BAS[h]	-0.035; 0.018<br>(-0.044- -0.025)	0.008; 0.012<br>(0.002–0.015)	-2.79	15′[m] PERG P50-N95 A[i]<br>6 M[g] minus BAS[h]	48.58<br><0.001	-7.08<br><0.001
15′[m] PERG P50-N95 A[i] (logμV)[l]<br>12 M[g] minus BAS[h]	-0.047; 0.021<br>(-0.058- -0.035)	0.013; 0.013<br>(0.005–0.020)	-3.41	15′[m] PERG P50-N95 A[i]<br>12 M[g] minus BAS[h]		-9.73<br><0.001
15′[m] VEP P100 IT[e] (logms)[f]<br>6 M[g] minus BAS[h]	0.152; 0.078<br>(0.109–0.195)	-0.033; 0.054<br>(-0.064- -0.002)	2.74	15′[m] VEP P100 IT[e]<br>6 M[g] minus BAS[h]	367.34<br><0.001	4.33<br><0.001
15′[m] VEP P100 IT[e] (logms)[f]	0.200; 0.100	0.127; 0.189	0.49	15′[m] VEP P100 IT[e]		-24.89

12 M[g] minus BAS[h]	(0.146–0.253)	(1.157–1.375)		12 M[g] minus BAS[h]		<0.001
15′[m] VEP N75-P100 A[i] (logμV)[l]	-0.038; 0.044	0.016; 0.016	-5.01	15′[m] VEP N75-P100 A[i]	10.34	10.34;
6 M[g] minus BAS[h]	(-0.062- -0.014)	(0.007–0.025)		6 M[g] minus BAS[h]	<0.001	<0.001
15′[m] VEP N75-P100 A[i] (logμV)[l] 12	-0.036; 0.044	0.008; 0.017	-3.57	15′[m] VEP N75-P100 A[i]		-3.52
M[g] minus BAS[h]	(-0.062- -0.0137)	(-0.0017- –0.018)		12 M[g] minus BAS[h]		<0.001
15′[m] RCT (logms)[f]	-0.039; 0.039	0.007; 0.018	-3.07	15′[m] RCT	13.30	-3.68
6 M[g] minus BAS[h]	(-0.061- -0.018)	(-0.004- -0.017)		6 M[g] minus BAS[h]	<0.001	<0.001
15′[m] RCT (logms)[f]	-0.062; 0.041	-0.001; 0.031	-2.03	15′[m] RCT		-4.84
12 M[g] minus BAS[h]	(-0.084- -0.039)	(-0.018–0.017)		12 M[g] minus BAS[h]		<0.001

[a] Cohen's d = Cohen's d values; [b] 1SD = one standard deviation; [c] CI = confidence interval; [d] 60′ = visual stimuli in which each check subtended 60 minutes of the visual arc, respectively; [e] IT = P100 Implicit time; [f] logms = logarithm of milliseconds; [g] M = Months [h] BAS = Baseline; [i] A= N75-P100 Amplitude; [l] (μV) = logarithm of microVolt; [m] 15′ = visual stimuli in which each check subtended 15 minutes of the visual arc; [n] ANOVA = One-way analysis of variance; [o] Tukey = Tukey comparisons method.
